# Blockchain protocols in clinical trials: Transparency and traceability of consent

**DOI:** 10.12688/f1000research.10531.5

**Published:** 2018-02-01

**Authors:** Mehdi Benchoufi, Raphael Porcher, Philippe Ravaud

**Affiliations:** 1 Département d'Epidémiologie Clinique, APHP, Paris, France; 2 Centre de recherche Inserm Epidémiologie et Statistique Paris Sorbonne Cité (U1153), Université Paris Descartes, Paris, France

**Keywords:** clinical trial, blockchain, smart contract, consent, e-consent, transparency, reliability, reproducibility

## Abstract

Clinical trial consent for protocols and their revisions should be transparent for patients and traceable for stakeholders. Our goal is to implement a process allowing for collection of patients’ informed consent, which is bound to protocol revisions, storing and tracking the consent in a secure, unfalsifiable and publicly verifiable way, and enabling the sharing of this information in real time. For that, we build a consent workflow using a trending technology called Blockchain. This is a distributed technology that brings a built-in layer of transparency and traceability. From a more general and prospective point of view, we believe Blockchain technology brings a paradigmatical shift to the entire clinical research field. We designed a Proof-of-Concept protocol consisting of time-stamping each step of the patient’s consent collection using Blockchain, thus archiving and historicising the consent through cryptographic validation in a securely unfalsifiable and transparent way. For each protocol revision, consent was sought again.  We obtained a single document, in an open format, that accounted for the whole consent collection process: a time-stamped consent status regarding each version of the protocol. This document cannot be corrupted and can be checked on any dedicated public website. It should be considered a robust proof of data. However, in a live clinical trial, the authentication system should be strengthened to remove the need for third parties, here trial stakeholders, and give participative control to the peer users. In the future, the complex data flow of a clinical trial could be tracked by using Blockchain, which core functionality, named Smart Contract, could help prevent clinical trial events not occurring in the correct chronological order, for example including patients before they consented or analysing case report form data before freezing the database. Globally, Blockchain could help with reliability, security, transparency and could be a consistent step toward reproducibility.

## Introduction

Patient participation is the *sine qua non* condition for clinical trials to occur and for medical research to improve ^1, 2^ ( http://www.mc.vanderbilt.edu/crc/workshop_files/2011-09-09.pptx). However, in practice, the informed consent process is difficult to handle in a rigorous and satisfactory way. The US Food and Drug Administration (FDA) has reported on the frequency of clinical investigator-related deficiencies, showing that almost 10% of trials they monitored have issues with consent collection, such as failure to re-consent when new information becomes available; failure to provide copies of the document to subjects; use of incorrect, expired consent forms or non-validated, unapproved forms; consent forms not signed or not dated; missing pages in consent forms provided to participants; failure to obtain written informed consent; parental permission obtained after child assent; and changes made to consent forms by hand and without Institutional Review Board (IRB) approval ^3^ ( https://your.yale.edu/sites/default/files/commonproblemsininformedconsent_2013_vf.pptx). 4 documents fraud in clinical trials such as issues of backdating consent documents. Examples of such misconduct were reported by the FDA in 2007 ^5, 6^ regarding the clinical trial of the safety and effectiveness of oral telithromycin and amoxicillin/clavulanic acid in outpatients. The most commonly fabricated documents are patient diaries and informed consent forms ^4^.

The FDA noted a global trend in the decrease in frequency of these issues in their investigations: in comparing 2000–2009 to 1990–1999, the frequency of issues related to the consent process in reviewed clinical trials were reduced by a factor 4 ^7^. However, the study by Seife ^8^ questioned the FDA results ^9^. The authors analysed hundreds of clinical trial FDA inspection documents, covering 1998–2013, and showed that a substantial number presented evidence of research misconduct; 53% were related to failure to protect the safety of patients and/or had issues of oversight or informed consent.

This situation can lead to dramatic events, as occurred in France in January 2016: a trial testing BIA 10-2474 as an analgesic caused the death of a participant. The French inspection agency Inspection Générale des Affaires Sociales seemed to prove that re-consent was not sought after major neurological side effects occurred in some patients, which led to participants being included in the clinical trial without being informed of this issue and still receiving doses of the analgesic ( https://fr.wikipedia.org/wiki/BIA_10-2474, 2016.09.05 version). Another example in the United Kingdom occurred when a general practitioner was struck off after testing drugs on patients who did not give their consent ^10^. A recent, popular case of serious scientific misconduct involved the DECREASE studies performed by Don Poldermans. The results of these studies were invalidated and some related publications retracted because among many other frauds including data invention, informed consent was not proved to have been obtained before the randomised controlled trials were implemented ( https://en.wikipedia.org/wiki/Don_Poldermans, 2016.09.05 version; http://retractionwatch.com/category/by-author/don-poldermans/).

Obtaining an individual’s consent is strictly tied to the Helsinki declaration ^11, 12^ ( https://www.wma.net/policies-post/wma-declaration-of-helsinki-ethical-principles-for-medical-research-involving-human-subjects/), which provides the good practices that any stakeholder conducting a clinical trial should follow. Point 26 of the Declaration states that each participant should be informed of the aim, methods, sources of funding, conflicts of interest, affiliations of the researchers, anticipated benefits and risks, and post-study provisions, and these conditions must be met to obtain freely given informed consent. In practice, regulation agencies, such as the FDA, provide recommendations and mandatory commitments for consent to be collected under the right conditions ^13^ ( http://www.fda.gov/downloads/RegulatoryInformation/Guidances/UCM405006.pdf). Among these recommendations, and of major importance, informed consent should be documented by a signed and dated written consent form, which is particularly meaningful with Blockchain technology.

In addition, consent collection is a dynamic process; it is not a one-shot process ending when consent is sought before a clinical trial begins. As explained by Gupta ^1^, there are circumstances under which consent has to be sought again from a participant, corresponding to any time the trial protocol is revised extensively. This is a fundamental fact when ensuring patients’ rights and transparency of a clinical trial ^14, 15^. Indeed, as detailed in some IRB guidelines ( http://www.irb.pitt.edu/sites/default/files/reconsent guidance.pdf; http://www.mayo.edu/research/documents/29-re-consent-or-notification-of-significant-new-findingspdf/doc-10027714; https://your.yale.edu/sites/default/files/reconsentingprocedure_1.pptx), there are many situations in which patient re-consent must be sought or patients should be notified of trial minor issues, such as new risks, significant changes in the research procedures, and worsening of the medical condition. Documents that are to be sent to patients can be consent-form addendums, an information letter or a fully revised consent form. Of course, the revised consent form must be approved by an IRB. The FDA has highlighted the need to conceive a mechanism that ensures that the most recent revised consent form is in use in a clinical trial and stipulates that time-stamping is such an approved mechanism ^12, 13^ ( http://www.fda.gov/downloads/RegulatoryInformation/Guidances/UCM405006.pdf).

**Figure 1.**
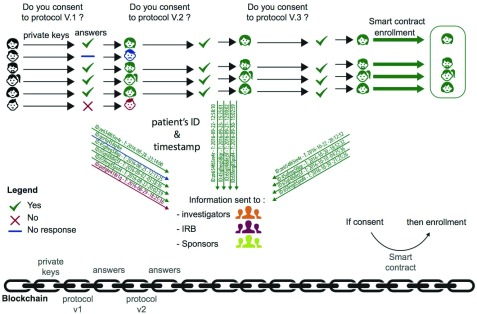
Consent collection Blockchain workflow.

As indicated in Figure 1, consent is a dynamic process that involves a complex circuit of data and interacting actors and should involve retaining all information about this on-going process, for example, what participants were notified, when the notifications were delivered, which of the participants consented or re-consented, and when the participants consented or re-consented. This information should circulate among the clinical trial stakeholders in real time.

Blockchain is a new technology, a giant shared datastore, stored in a secure and decentralised way (see Sotoshi Nakamoto seminal paper, https://bitcoin.org/bitcoin.pdf). It is widely considered a core infra-structure of digital assets about which we want to ensure reliability, powering a wide range of services by transparency and traceability. In this context, the Blockchain emerging and promising technology ( https://en.wikipedia.org/wiki/Blockchain_(database)) can bring a solid basis for transparency of the enrolment phase to all stakeholders of a clinical trial, especially in the context of obtaining participant consent. Three core functional principles of this technology can play a fundamental role, as follows:

1. Unfalsifiable time-stamping information: that is, proof of existence of any piece of data. When stored, these data are provable and immutable via a strong cryptographic protocol. Moreover, these proofs of existence can be checked on a public website ( https://opentimestamps.org, https://arxiv.org/abs/1502.04015). This transparency is of interest to any stakeholder.
2. Smart contract: a contract that is algorithmically implemented and binds any change in the protocol to patient consent needing renewal.
3. Decentralized nature of the protocol: gives to the patient, or more widely to patient communities, control over their consent and its revocation. The end-to-end connectivity creates a network that empowers patients and researchers as peers.

The current implementation is an application of the first principle. Ideally, we must build a patient authentication system that does not rely on any trial stakeholder so that we can benefit from the decentralised and trustless nature of the Blockchain network.

In a broader clinical trial setting, with the secure time-stamping functionality, Blockchain can directly help prevent an a posteriori reconstruction of endpoints or outcomes in clinical trials ( http://www.bgcarlisle.com/blog/2014/08/25/proof-of-prespecified-endpoints-in-medical-research-with-the-bitcoin-blockchain/).

### Blockchain comes into play

***At a clinical trial level.*** Blockchain technology can act as a SafeGuard for the complex and wide range of actors required in clinical trials. In practice, the proof of existence for consent is time-stamped and stored in Blockchains, thereby enabling clinical research stakeholders, such as sponsors, investigators and IRBs, which can be numerous in multi-center clinical investigations, to share consent– and re-consent–related data in real time and can archive and historicise consent sets, which can be matched with each revision of the protocol.

***At a patient level.*** Implementing a “privacy by design” technology and securely and transparently archiving any dataset that needs to be stored is a substantial step toward improving enrolment phase methodology. Of course, there are ways to achieve a certain level of security in data archiving. Namely, Distributed Ledger Technologies allowed for such security before Blockchain was invented, but the breakthrough in its validation protocol, called “proof-of-work” in the Bitcoin network, allowed for the design of an open inclusive system whereby peers contribute to the network effort to validate transactions, so-called “mining”. Moreover, drawing on ways to securely and transparently collect informed consent, being careful with participant rights, and so empowering them, could improve the enrolment rate. Indeed, the participation rate in clinical trials remains weak ^14^. A systematic review comparing different enrolment techniques showed that among several other explanations, collecting patient consent in an open and secure way was best at improving the rate of enrolment ^14, 16^.

## Methods

In this proof-of-concept (POC) study, we enrolled 27 volunteers from among the staff of the Clinical Epidemiology Department at Hospital Hôtel Dieu (Paris, France). We had no exclusion or inclusion criteria. We ensured that each of the volunteers had email access.

We designed a fake experimental clinical trial protocol to compare the effect of “cisplatin versus ledgerlin”, the last substance being a neologism derived from the critical public datastore shared by all Blockchain users called “ledger”. The protocol was accompanied by a consent form that mimicked a design used routinely.

Each of the to-be-enrolled users was assigned a private key to sign data and documents, and in practice this would be used to publish their signed consent, which was to be anchored to the Blockchain.

### Blockchain networks

Examples of Blockchain networks are Ethereum ( https://www.ethereum.org), Bitcoin ( https://en.wikipedia.org/wiki/Bitcoin_network) and Hyperledger ( https://www.hyperledger.org). For our purpose, transactions and their validations were run on the Bitcoin network because of the stability and immutability of the Bitcoin Blockchain with its large mining network, and the Application Programs Interface (API) it provides facilitates development. Moreover, it is the more widely used Blockchain network; thus, a relatively dense community of developers enables efficient support ( https://bitcoin.stackexchange.com). The front-end and back-end technologies that are detailed hereafter were implemented by using a Blockchain solutions provider, Stratumn SAS ( https://stratumn.com/).

**Figure 2.**
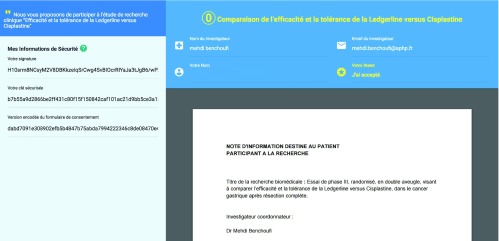
Patient web interface for Blockchain consent collection website.

A website was developed with a simple one-page interface ( Figure 2). On the front-end, it displayed the consent document, a checkbox verifying that the protocol was read and understood, and a push button that triggered the consent process.

In practice, the online signed document contained a piece of code called “Chainscript” ( http://chainscript.io), which contained all the critical information about the user, and the version of the protocol attached to the signature. Each proof of signature had a manifest that takes the form of a “hash” that is the digital proof of signature.

**Figure 3.**
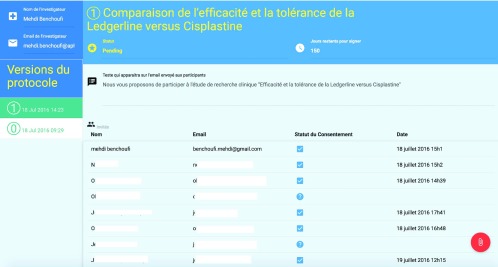
Investigator dashboard for Blockchain consent collection website.

On the back-end, pressing the “consent button” triggers Blockchain transactions: the proof of signature is time-stamped and stored in the Blockchain. These signatures were shared in real-time with a restricted group of individuals, namely the authors of this paper, who represent, in a real-life implementation, investigators, IRBs or sponsors. This group had access to a dashboard ( Figure 3) with the following: an administrator panel displaying the consent status of each user, the protocol that transparently stores the public keys of each consenting user (through Chainscript), and the history of each released version of the protocol and the consent and re-consent of the user attached to each of the versions.

### Authentication method

For each user, a pair of private-public keys were provided ( https://msdn.microsoft.com/fr-fr/library/windows/desktop/aa387460(v=vs.85).aspx). These are asymmetric cryptographic data that enable authentication on Blockchain. These were randomly attached in one-to-one correspondence to the user’s emails. We focused on Blockchain’s usage in the time-stamping and archiving logic. We did not let users create or use their own Blockchain authentication setup (i.e., if a user owned a Bitcoin account, the key and Bitcoin address were not allowed to be used). This restriction was related not to the Blockchain complexity but rather to maintain a simple and common email-focused authentication process. Other ways for authentication include the physical devices USB keys or cell phone fingerprints, but this would have been outside the focus of our protocol-related problematics.

### Workflow

The process was as follows:

- The study investigators send an email to the user.
- The user receives the email, which contains a web hyperlink redirecting them to the web interface that displays the consent form.
- In the background, after clicking the consent button without the user experiencing any Blockchain transaction-related complexity, the user signature is registered in the Blockchain and time-stamped.
- Each time the protocol is updated, investigators send an email explaining the major changes that occurred and users are invited to sign the revised consent form.
- Each step of this process is updated on the investigators’ administrator panel with the version of the consent form and the user's consenting status.

### Proof of existence - Chainscript

Signatures of the evolving consent document were registered on the Blockchain. Moreover, all of the relevant interactions of the user with our platform were stored in the Blockchain (i.e., the consent form uploaded by the investigator; email requests to users, and participant signatures) according to the Proof-of-Process concept developed by Stratumn, a method for proving the integrity of a process between partners ( https://stratumn.com/pdf/proof-of-process.pdf).

The piece of data verifying and synthesising all this information is called a Chainscript. It is a JSON formatted data structure holding all the information related to the protocol and users’ consent. Chainscript was developed by Stratumn SAS, especially dedicated to attest the steps in trusted workflows ( http://chainscript.io/). Chainscript is an open standard. The philosophy behind it is to be able to prove the integrity of a process with a single JSON data structure by securing the who, when, what and where of a sequence of steps that are linked in chronological order. Each sequence corresponds to a segment, and each segment holds some metadata, an identifier called a hash, and a pointer to the preceding segment. The critical information maintained in Chainscript are the hashes, which are the proofs of the existence of data. Since each Blockchain transaction has a cost, we grouped the transactions. With Chainscript, a series of Blockchain transactions can be wrapped into the same logic flow, thereby preventing too-intensive requests to the Blockchain network, which can be costly.

With this information, we need to check the proof of specific data after they are merged. The Chainscript solution relies on a singular data structure, a Merkle Tree, which in a way “hashes the hash”, preserving in one single hash all the hashes, so that if any hash is corrupted, the entire tree is invalidated. For a wider perspective of this JSON file, Chainscript stands as a “proof-of-process” document. The process one wants to track through Blockchain is represented by structures called Chain Maps; the events in the process are represented as Chain Segments, each of which holds a reference to a previous event. The transition from one event to another or from one state to another in the language of state machine is executed by scripts called Chain Scripts. The Chain Script describes in a homogeneous, transparent and checkable way all the history of the process with its constitutive events. This document holds every proof of data and is checked against Blockchain transactions on dedicated public websites. In our implementation, the Chainscript code is held in the PDF consent form, storing its hashed content, all its versions (corresponding to protocol revisions) and all the signing users for each version. Chainscript can be publicly verified without any proprietary tool.

A positive side effect of tracking each step of a user’s interaction with the platform is that exhaustively storing the data enhances the barrier to fraud.

## Results

### Clinical trial master document

We collected consent and re-consent for users and stored them in a transparent, unfalsifiable, verifiable way. These data were encoded in a single document. This document holds the stakeholders’ identifiers, the users’ identifiers, time-stamps of the protocols being sent, consent status, time-stamps of the consent, and the version of the protocol to which the consent is attached.

This master document was shared in real-time by key actors who oversaw the POC study. It was registered in the Blockchain safely, so that this group of stakeholders retained the time-stamped proof of the consent status in an immutable document. We stress again that this document is incorruptible, and its consistency can be checked on any dedicated public website (e.g., a website that enables checking of Bitcoin transactions), thereby proving the correspondence between each version of the protocol and its related consent.

Technically, these data are synthesized in the open data format Chainscript as follows:


"segment": {



  "link": {



    "state": {



      fileName: "protocole.pdf";



      uploadedBy: "investigateur";



    },



    "meta": {



      "title" : "Protocole",



      "tags" : ["POC", "Essai clinique", "Hôpital Hôtel-Dieu"],



      "priority": "0"



      "updatedAt": 1455532426303,



      "mapId": "56c11d0ea55773bc6e681fde",



    }



  },



  [{ "Document_ID": "NOTE D INFORMATION DESTINEE AU PATIENT.pdf"",



   "Doctor_Name": "***",



   "Doctor_Email": "***@aphp.fr",



   "PDF_Title": "",



   "Conditions": "",



   "ExpiresIn": "XX",



   "Max_Patients": "XXX",



   Invites:



   [{



   "Email": "***@aphp.fr",



   "Name": "***",



   "Address": {



    "hash": "2568ce846c1391d94065df6cc4a42720369bcec9",



    "type": "pubkeyhash",



    "network": "livenet"



    }



 },],



 },[



       "signature",



   "***",



   "***@aphp.fr",



   0,



    "H6qy9U3S+BNqreKwMgEnDAHij3wNMcq4T2+X9axzx65Zd+HDy16tr03YPT4oKkGtW820so0D+0Pk2UTrwnXiLKs=",



    {



        hash: "6786ce716b2ac8e14b20e0a2fd8b88a7994d4a10",



        type: "pubkeyhash",



        network: "livenet"



    },



    "2016-07-08T13:11:15.824Z",



    "method__saveSignature"



],[{



    "DateSigned": "2016-07-08T13:11:15.824Z",



    "Signature":         "H6qy9U3S+BNqreKwMgEnDAHij3wNMcq4T2+X9axzx65Zd+HDy16tr03YPT4oKkGtW820so0D+0Pk2UTrwnXiLKs=",



    Consent: 0



    }]


All this information is bound together in one data structure, so that the whole set of obtained consent, with the uniquely attached version of the protocol, forms an immutable global data. Changing a single element breaks the entire data structure.

Asserting that these data are publicly verifiable means that any alleged document with a claim of consent bound to emails and protocol versions can be verified by “hashing” it with the public key and then comparing it with some transaction inside the public Bitcoin Blockchain by hand, by downloading the Blockchain (but this is a major undertaking because the Bitcoin Blockchain size at the time of this writing was > 160 Gb) or by using some public website that offers transaction validation services, such as https://blockchain.info.

In the interest of user confidentiality, the master document cannot be made available.

### Technical details of the POC

During the study, we sent two versions of the protocol ( Supplementary File 1 and Supplementary File 2) with which we sought users’ consent; each consent was attached to a specific version of the protocol.

Users were given a digital signature and secured key, each consisting of a hash. Among the users of this experimental study, 14 gave their consent to the two versions of the protocol, 9 gave their consent to only one version of the protocol and 2 did not give consent at all and 2 did not respond to any consent form.

The interaction of the user with the online interface, namely accepting or refusing to give consent, led to a transaction validated in Blockchain. Each version of the protocol had a unique identifier, called a hash. The hash was uniquely attached to the content of the protocol document. The correspondence between the consent document and the hash is one-to-one; namely, if one single letter is changed then the hash is broken.

**Figure 4.**
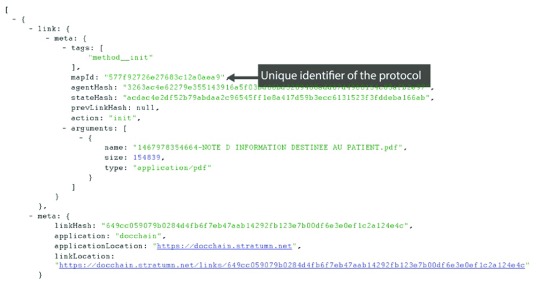
Consent collection master document: unique identifier protocol.

**Figure 5.**
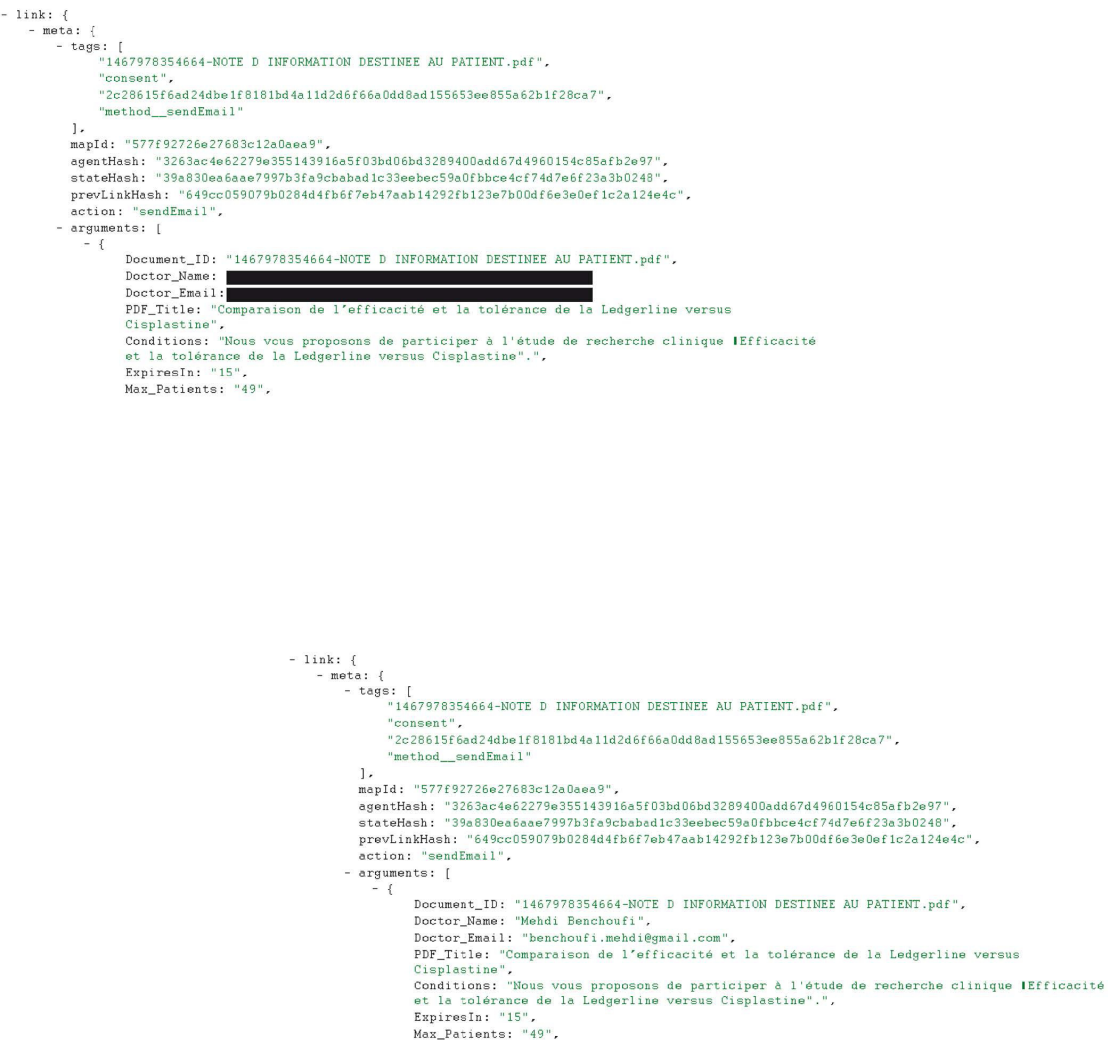
Consent collection master document: investigator identifiers.

**Figure 6.**
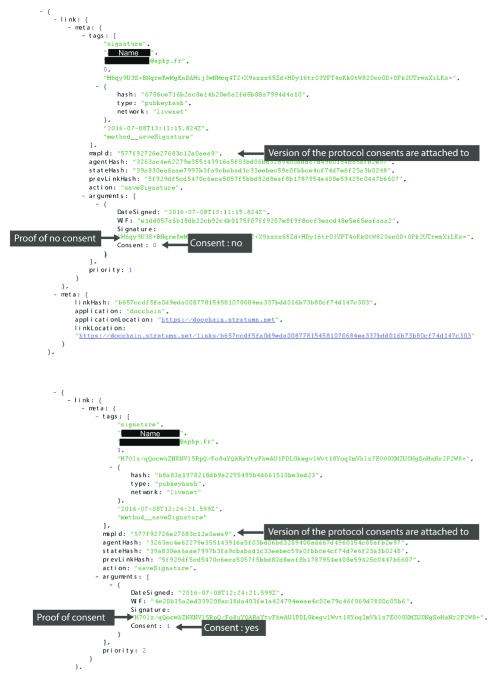
Consent collection master document: consent status bound to protocol revision versions.

Figure 4 shows the identifier of the protocol document highlighted in the Chainscript master document. Figure 5 displays the investigator identifiers, and Figure 6 reveals the consent status bound to the protocol revision version.

## Discussion

In a context of growing mistrust of institutions and extreme sensitivity about rights to information and respecting privacy, we need to build a consent process as secure as possible with the current state of knowledge and technologies. Trust is the critical pivot to engage subjects in clinical trials. The Blockchain technology was specifically designed to ensure trust.

Blockchain is an emerging, fast-evolving technology. The intense atmosphere around the development and use of Blockchain is similar to technology development during the early stages of the Web: it took several years before formatting such as html or css became Web standards. Blockchain technologies are gaining increasing attention, and Blockchain networks are proliferating; examples are Ethereum, Bitcoin, Hyperledger or private Blockchain networks. Which of these will be the Blockchain network standard is not clear or even if there will be unique standard. Public networks are interesting because of the idea of a community-driven approach and scalability, but private ones can offer a certain level of customization.

We chose Bitcoin rather than Ethereum because even though Ethereum allows for implementing what we achieved through the Bitcoin networks, in our current implementation, we grouped transactions in order not to require transaction fees at each user step and in view of a real-life clinical trial, in which users will manage their own pair of keys. Therefore, we used a Bitcoin feature allowing for multiple inputs and signing transactions through multiple signatures, also named "multisig". There is no such a feature of multiple inputs in Ethereum, but there is a workaround. We would have used two contracts, the first receiving the transactions to queue them and depending on some threshold, an amount of Ethereum crypto-currency (ETH) or an amount of transactions. The first contract would hit a second contract that would register the proof of data, but the process might be complex.

Alongside this fast-developing technology, there are still some infrastructure obstructions that need addressing, namely, delays in transaction validation. On the Bitcoin network, the validation process (via so-called mining) takes about 10 minutes ( https://www.quandl.com/data/BCHAIN/ATRCT-Bitcoin-Average-Transaction-Confirmation-Time). In the present study’s context, we are not tied by real-time requirements measured in seconds, so it is not a major obstruction. Moreover, the ChainScript logic we implemented in our POC allows for grouped-network request validation, which prevents the Blockchain network from computation overload and allows for scaling our method to a large patient cohort. For an idea of the transaction costs, the fees of the Bitcoin transactions vary — at the time of writing, between 0.0015 to 0.0016 BTC, depending on the priority of the transactions, which corresponds to about 10€. However, in our implemetation, we used a special data-structure called a Merkle tree to group the data we wanted to store as proof-of-evidence. This tree, whose critical element is the root, the Merkle root located at the top of the tree, can hold hundreds of thousands of hashes, so that validating the transactions by batches of 10.000 or even more hashes helps control the cost of the Bitcoin network requests to some milli-bitcoins. We refer the reader to https://live.blockcypher.com/btc/ to check the cost of the Bitcoin fees. This matter is of special interest knowing that Bitcoin currency is quite volatile: indeed, the price of this currency has increased from about 1000$ at the beginning of 2017 to 15.000$ at the beginning of 2018. The cost of transaction fees affects the transactions we can initiate. However, we can mitigate this cost with a data structure that helps absorb in part the costs of the transactions. Indeed, some flexible data structures can be fed with more elements to group them for the Blockchain network and validate them together. We could size the Merckle tree to host a batch of 100,000 hashes and validate them in a single transaction so that we have some leverage when the Bitcoin currency is too high. However, the current transaction fees, about 9 USD, at the time of writing (29 January 2018) and constraints of time when waiting for the Merkle tree to be entirely filled when the number of its leaves becomes large are issues. Of course, reasonning at worst and supposing that we do not group the transactions and that we trigger one transaction for each individual consent, a cost of 10USD remains negligeable with regards to the total cost of clinical a trial. So, as long as the fees remain at this level, this should not be a subject of major concern, but, regarding future upward volatility, let's mention that currently, there are major technical efforts in implementing Blockchain solutions, which could lead to a better resilience of transaction fees to crypto-currency volatility. 

More generally, to tackle this challenge of scaling the network, with a massive amount of transactions, there are some implementations of Bitcoin-based protocol isolated from the Blockchain; the most renown is called SideChain ( https://www.deepdotweb.com/2014/06/26/sidechains-blockchain-2-0/; https://www.reddit.com/r/Bitcoin/comments/2kdxy8/). In deploying “energy-savvy” solutions that could reasonably absorb the costs of an important amount of transactions with a large clinical trial, some Blockchain implementations are based on Proof-of-Storage rather than Proof-of-Work, the latter being the cryptographic problem that nodes have to be solved in order to validate a transaction, which is extremely power-intensive. In a Proof-of-Storage network, nodes agreeing to store files allows for validating transactions.

Moreover, in terms of the authentication process, when the use of Blockchain technologies becomes more common, users may already possess a Blockchain public-private-key identity. Therefore, sending keys for access and identification later in the signing process will be obsolete. This situation will require maintenance of a double key attribution (as explained in the “Authentication method" section) for users who do not have any Blockchain network identity and to be able to account for those who already have one. In the latter case, the digital signature of these users will need to be verified.

The resilience of the network to malicious attacks is a vast subject and draws intensive interest by the whole Bitcoin developer community. Basically, the first attack is an attacker controlling multiple nodes to try to solve the Proof-of-Work problem, thus increasing the probability of gaining more mining coins. Unfortunately, this “Sybil attack” would fail because the difficulty of the problem increases with the number of nodes. A more substantial attack, at least among the Blockchain developers, is the well-known 51% attack, whereby an attacker gains more than a half of the network computing power, giving the authority to control block addition. However, even in that case, the attacker will not be able to corrupt data or steal money because it requires the private key attached to the Bitcoin account, and this attack will never occur successfully on the Bitcoin network. Even if it were the case, “double spend” will lead the non-accomplice nodes to distrust the Blockchain network. Also, in terms of attacks, because Blockchain is a shared database, anyone has a copy and no data will be missing or corrupted. We refer the reader to a thorough treatment in https://en.bitcoin.it/wiki/Weaknesses detailing the potential attacks with Blockchains.

One step further, we can schematically consider two main issues regarding the consent process, the first related to the quality of the process itself and the second to the identity of the individual consenting. We focused on the first point and tackled the issues raised by the FDA ^3^. Indeed, in this POC study, we considered problems in which existing patients were included in a study in the presence of their physician or staff so that ensuring that the consenting participant was precisely the one expected was not a critical matter. In terms of the issues reported in the literature and by regulatory bodies, binding the hashed protocol and its versions to the consent, preventing from backdating and giving not only a time-stamped but a trusted consent, gives more strength to the consent process, which is what we were looking for in this POC. Moreover, in the setting of a real online consent process, a patient who would not effectively consent (e.g., if there were some fraudulent operation registering him/her as a consenting participant) would not actually participate in the study. However, in this situation, even if the patient would not show up , the data subject to the Blockchain transactions would be stored and therefore be false. Because the Blockchain is endowed with a data-incorruptibility quality, there is no way to go back. We could put some level of dissuasive control of these situations by sending an email to the user who allegedly consented or revoked consent, but this would be better done through a Smart Contract to ensure the integrity of the process. Any patient protest would then lead to ignoring this Blockchain transaction.

Here we emphasize two points. First, data invention issues do not fall into the scope of misconducts easily avoidable by Blockchain technologies, and this kind of falsification described is difficult to control, although we think that Blockchain-based systems feature a higher barrier to entry of such practices (generating key pairs, credible sequence of time-stamped events, hiding the IP of web-requests). Second, the solution we propose leads to improvement with respect to the current state of practices and can be directly implemented, provided that some entity holds the responsibility of key-pair generation, so that among stakeholders such as investigators, sponsors, IRBs, the FDA or EMA, we could delegate this responsibility to some most trusted entity, under the supervision for instance of a regulatory agency.

A subject of concern is the issue related to asserting identity, which will be of importance in the context of a real clinical trial and should be done in a more secure manner than linking between a participant and his/her digital identity via an email address. In a production application, we could implement several solutions to secure the digital identity of participants, at least implementing a Know Your Customer (KYC)-like solution to link digital identities and physical entities. KYC solutions are techniques used by fiscal administrations or banks to secure their online services. Another way could involve a Blockchain-based solution to provide material objects such as USB keys, holding the cryptographic signatures, which can be unlocked by an easy-to-remember code.

In regard to the interplay between individual identity and effective consenting participants, an online solution could be designed using Smart Contract, considering that the major forgery can come from a malicious party trying to consent on behalf of a user. This would imply a shift to some Blockchain such as Ethereum. So, we could generate key pairs coherently from some token provided by the investigator, enabling stakeholders to link the public key attached to transactions and the identity deduced from the token; every transaction would then hit a Smart Contract triggering an email informing the user of the transaction, here for instance the consent status. However, even if this is hacking around the problem, the current key management side does not bring a satisfactory solution to subject identification and is a limitation of our current implementation.

In a context where patients master the key generation process and applying the same process we detail in this POC, we would be close to attaining a trustless consent process that promotes the patient community as a decisive actor of clinical trials. The literature documents barriers to enrolment, especially when barriers are strongly related to community or ethnicity-related issues ^17, 18^. The decentralised, transparent and secure nature of a Blockchain protocol may meet the conditions for improved engagement of patient communities in clinical trials. It could help optimize patient enrolment and in turn, through a more transparent and trusted process, create a bridge between clinical research teams and patient communities. The latter are novel incomers in our digital age and their commitment is critically dependent on building clinical trials as a highly trusted process.

We did not implement a consent revocation workflow. However, there is no issue in transposing the Blockchain transaction logic we implemented for the consent for this purpose. However, we should be careful about the fact that if the consent or its revocation can be given or withdrawn with no problem, these actions cannot be erased from the Blockchain. Indeed, if participants revoked their consent by accident, then the action can be reversed, but data containing the revocation of the consent and the cancellation of this revocation will remain.

From a technical point of view, implementing a Blockchain-based solution will not be difficult to integrate in standard data management systems because the core of the process supposes the “notarization” of consent. This core can be wrapped up as a plugin or even more simply remotely accessible from so called APIs. To make the process as reproducible as needed, we refer the reader to a “Technical guide to installation.md” markdown file ( Supplementary File 3) where we detail the main element for the experiment to be reproduced, and we indicate for developers useful resources to implement their own solution. From a user point of view, we consider our current solution almost ready-to-use in a production setting. Indeed, the Blockchain complexity is totally hidden from the interacting user while benefitting from Blockchain functionalities. In practice, there is no burden in the front-end website interactions, although first, users should be informed that technical tools are used to ensure transparency, security, and veracity of the consent process, and second one should complete the implementation we propose by some additional user interface to check the correct understanding of the protocol by the user. A quiz at the end of the protocol reading could be interesting. However, the latter point is related to online nature of our consent process rather than Blockchain.

In the range of more prospective considerations, obtaining consent must be a “lock” before participant inclusion in clinical trials, so that investigators will not be able to include a patient in the trial until consent is collected. To ensure a strict parity between enrolled patients and included patients, we could use a tool along with Blockchain called the Smart Contract ( https://en.wikipedia.org/wiki/Smart_contract, 2017.05.26 version). This piece of code holds a programmatically written contract between as many parties as needed, without any third-party, and executes algorithmically according to the terms provided by the contracting parties. In our context, a Smart Contract could be built to execute with the only condition that patients will only be included when the enrolment is complete. Technically, every Blockchain transaction can have a lock associated with it, and transactions can be pending and triggered at an agreed-upon contract time. For example, the signature of the consent would trigger the execution of a Smart Contract that would unlock the edition of an electronic case report form.

Health is entering a Big Data era, with 2.5 exabytes of data produced each day, 50 billion devices expected by 2020 ^19^ and 500 billion by 2030. As well, objects may be connected to the Internet ^20^, so that online-based clinical trials will represent a substantial part of clinical research. In this expectable context, enforcing and consolidating the online consent process, as explained here ^21^, can be harmed if conditions of trust are not met ^22^. Blockchain could be an interesting tool to ensure the quality and security of the process.

Finally, the empowerment of Blockchain users in that they participate in a network that does not depend on a trusted third party has to be balanced. Indeed, patients may have some level of control by using a standard written consent process. However, as pointed out by the FDA, this process has a number of issues, and a recent study pointed out that 80% of surveyed participants would prefer an online consent process, which has some consequence on the selection bias related to complex written consent forms with low adherence ^23^. So, considering the consent process as a whole, collecting consent by a blockchain process may affect the trust patients may have in these protocols, because the registered proof-of-data could leave an indelible trusted digital trace. This question of trust is especially important, at the time societies are involved in debates about patient rights and privacy at the same time of mistrust toward public authorities and institutions. However, again considering the consent process as a whole, the empowerment would be commensurate with each consenting subject participating in the control of the system, and so are peers on the Blockchain network. Our current implementation is limited by the key management that is not initiated by the end-user, so this limits the level of empowerment we can attach to the whole process.

However, Blockchain is certainly not a “one size fits all” solution to the problem of a low enrolment rate. Indeed, there are many other parameters that interfere with the enrolment, which fall beyond the scope of transparency, user control and reliability that Blockchain technology helps to achieve and include age, sex, cultural background, socioeconomic factors, lack of educational materials ^24^, readability and length of consent ^25– 27^, limited awareness about clinical research ^28^, patient–physician relationship ^29^ and momentum of consent request ^30^. Our method did not address the question of consent collected in singular situations, such as intensive care, unconscious patients or psychiatric pathologies. As well, the relation between patient engagement and Blockchain-driven consent is not direct but rather is mediated through the trust that Blockchain enforces. As previously explored ^31^, lack of trust of industry-sponsored clinical trials compromising consent, transparency about highly evolving technologies, such as artificial intelligence and their alleged or expected impact on healthcare puts trust at the high level of concerns in our increasingly informed societies. Blockchain was precisely a response to growing mistrust in institutions, historically addressed in the context of currencies and centralised bank systems. So, distributing pieces of trust through a network may help achieve more symmetric and transparent information. Deployed in the context of clinical trials, the first step of which is precisely the consent process, could invite patients to be more trustful of clinical trials and so engage more.

## Conclusion

Keeping track of consent collection is consolidated through the use of Blockchain technology. In this proof-of-concept study, all consent-related data can leave an unfalsifiable and verifiable fingerprint on the Blockchain. This is important both from the stakeholder’s viewpoint, letting them prove the existence and the consistency of the data, and on the patient’s viewpoint, giving them more visibility, transparency, and hence control over their consent.

Moreover, although not the focus of this paper, Blockchain technology, in that it does not rely on trust in a third party but inversely empowers peer-to-peer users by granting them control over consent agreement and revocation, can help gather conditions of improved privacy-respected freely given consent. Besides, given its decentralized protocol, it can help introduce communities to contemporary clinical research, opening, for clinical research, the path to implementing community management techniques to enroll patients by using a more targeted approach.

From a global perspective, the application of Blockchain technologies in the context of clinical research is broad and promising. Indeed, tracking the complex data flow with numerous diverse stakeholders and documenting it in real-time through a time-stamping workflow is a key step toward proving data consistency and inviolability and will therefore improve clinical trial methodology.

## Software availability

Latest source code available at: https://github.com/benchoufi/DocChain

Archived source code as at time of publication: doi, 10.5281/zenodo.237040 ( https://zenodo.org/record/237040#.WHSxorYrI_V)

Licence: 3-clause BSD licence

## Supplementary material

Supplementary File 1: Protocol and consent form (versions 0 and 1) used in the proof-of-concept study (in zipped file) (in French).

Supplementary File 2: Protocol and consent form (versions 0 and 1) used in the proof-of-concept study (in zipped file) (in English).

Supplementary File 3. Technical guide to installation.
