# Supplementary material for: Blockchain protocols in clinical trials: Transparency and traceability of consent
Source: F1000Res. 2018 Feb 1;6:66. Originally published 2017 Jan 23. [Version 5] doi: 10.12688/f1000research.10531.5 (PMC5676196; doi:10.12688/f1000research.10531.5)
Supplement: Supplementary file 1 [file f1000research-6-15062-s0000.tgz › 2ae80474-0dca-4f19-ba4b-5c59d9b49877.zip!/Supplementary File 1/Version 1/Protocol.pdf]

NOTE D'INFORMATION DESTINE AU PATIENT
PARTICIPANT A LA RECHERCHE

Titre de la recherche biomédicale **:** Essai de phase III, randomisé, en double aveugle, visant à comparer l'efficacité et la tolérance de la Ledgerline versus Cisplastine, dans le cancer gastrique après résection complète.

Investigateur coordonnateur :

Dr Mehdi Benchoufi

Coordonnées :
Hôpital Hôtel-Dieu
1 Parvis Notre-Dame - Pl. Jean-Paul II, 75004 Paris
Tél. : 01 42 34 78 15
E-mail : mehdi.benchoufi@aphp.fr

Promoteur :

Inserm CRESS - U1153 ; METHODS (Méthodes de l'évaluation thérapeutique des maladies chroniques)

Coordonnées :
Hôpital Hôtel-Dieu
1 Parvis Notre-Dame - Pl. Jean-Paul II, 75004 Paris
Tél. : 01 42 34 78 15

Avis favorable du Comité Consultatif de Protection des Personnes dans la Recherche Biomédicale de Paris le : 15/06/2016

Autorisation de l'Autorité Compétente (ANSM) le : 01/07/2016

Assurance : contrat n° 156, attestation n° 365
Conformément à la loi, le promoteur de cette étude a souscrit une police d'assurance auprès de MEDassurance.

Madame, Monsieur,

Nous vous sollicitons pour participer à une recherche biomédicale.

Nous vous présentons ci-dessous pour information, l'étude pour laquelle nous souhaitons votre participation volontaire. Nous vous laissons quelques jours pour réfléchir à cette proposition et restons à votre disposition pour répondre à toutes vos questions.

*__Contexte__ :

La Cisplastine, est la chimiothérapie de référence à la suite d'une résection complète en cas cancer gastrique. Des études précédentes, semblent suggérer que la Ledgerline serait plus efficace que la Cisplastine.

* **Objectif de la recherche:**

Evaluer l'efficacité de la Ledgerline sur la survie sans rechute en cas de cancer gastrique après résection complète. Evaluer la toxicité de ce médicament.

*** Méthodologie :**

Les patients inclus dans cette étude, sont des patients atteints d'un cancer de l'estomac ayant bénéficié d'une résection complète.
Suite à un tirage au sort les patients seront répartis en 2 groupes : un groupe recevant de la Cisplastine, un groupe recevant de la Ledgerline (randomisation).
Ni le patient, ni le médecin ne seront au courant du groupe dans lequel le patient a été affecté (double aveugle).

La durée du suivie sera de 4 ans. La surveillance se fera tous les 6 mois, elle comprendra un scanner thoraco-abdomino-pelvien, et un examen clinique.

Si au cours de l'étude, les analyses statistiques semblent suggérer qu'un des traitements est plus efficace que l'autre, l'étude sera suspendue et les patients pourront bénéficier du traitement semblant être le plus efficace.

*** Bénéfices attendus :**

Amélioration de la survie sans rechute.

*** Contraintes, obligations et restrictions imposées, inconfort, risques prévisibles :**

Les patients recevront 2 fois par semaine pendant 12 semaines l'une des 2 molécules, dans l'un des services d'oncologie participant à cette étude.

La Cisplastine et la Ledgerline, sont 2 molécules susceptibles d'entrainer des vomissements important. Un traitement antiémétique sera prescrit au patient dans ce cas.

La Cisplastine et la Ledgerline peuvent être responsables d'une chute des cheveux et d'une neutropénie.

* **Effets Indésirables**

Nous vous informons qu'une étude récente montre que la ledgerline peut être responsable de rares ulcérations œsophagiennes et d'exceptionnelles myalgies ou uvéite. De plus, des ostéo-nécroses de la mandibule ont été décrites lorsque le produit administré par voie intraveineuse à forte dose.

* **Frais médicaux**

Votre collaboration à ce protocole de recherche biomédicale n'entraînera pas de participation financière de votre part. Conformément à la loi, tous les frais liés à l'étude seront pris en charge par le promoteur de l'étude.

* **Confidentialité**

Toute information vous concernant recueillie pendant cet essai sera traitée de façon confidentielle.
Seuls les responsables de l'étude et éventuellement les autorités de Santé pourront avoir accès à ces données. A l'exception de ces personnes (qui traiteront les informations dans le plus strict respect du secret médical), votre anonymat sera préservé. La publication des résultats de l'étude ne comportera aucun résultat individuel.

Les données enregistrées à l'occasion de cette étude feront l'objet d'un traitement informatisé par le promoteur.
S'agissant de données nominatives, vous bénéficiez à tout moment, du droit d'accès et de rectification des données vous concernant auprès des responsables de l'étude et, en ce qui concerne les informations de nature médicale, ce droit est exercé par l'intermédiaire du Dr Benchoufi conformément à la loi 78-17 du 06 janvier 1978 relative à l'Informatique, aux Fichiers et aux Libertés, modifiée par la loi n°94-548 du 1er juillet 1994, relative au traitement des données nominatives ayant pour fin la recherche dans le domaine de la santé. Le projet a reçu un avis favorable de la CNIL en date du 15/06/2016.

* **Modalités de transmissions des résultats globaux de la recherche :**

Conformément à l'article L 1122-1 du Code de la Santé Publique (loi de Mars 2002 relative aux droits des malades les résultats globaux de l'étude pourront vous être communiqués si vous le souhaitez.

Vous serez informé des résultats de cette étude, dès qu'ils seront disponibles.

Ce projet est réalisé dans le cadre d'un financement par l'APHP et en partenariat avec l'Institut National du Cancer (INCa).

Nous vous laissons quelques jours pour réfléchir à cette proposition et restons à votre disposition pour répondre à toutes vos questions.

Après avoir lu cette note d'information, n'hésitez pas à poser toutes les questions que vous désirez à votre médecin traitant. Cette note d'information doit être conservée.

Nous vous remercions de votre coopération et, si vous êtes d'accord, vous demandons de bien vouloir signer le formulaire de consentement ci-joint.

FORMULAIRE DE CONSENTEMENT DESTINE
A LA PERSONNE PARTICIPANT A LA RECHERCHE

Titre de la recherche biomédicale **:** Essai de phase III, randomisé, en double aveugle, visant à comparer l'efficacité et la tolérance de la Ledgerline versus Cisplastine, dans le cancer gastrique après résection complète.

Investigateur coordonnateur :

Dr Mehdi Benchoufi

Coordonnées :
Hôpital Hôtel-Dieu
1 Parvis Notre-Dame - Pl. Jean-Paul II, 75004 Paris
Tél. : 01 42 34 78 15
E-mail :mehdi.benchoufi@aphp.fr

Promoteur :

Inserm CRESS - U1153 ; METHODS (Méthodes de l'évaluation thérapeutique des maladies chroniques)

Coordonnées :
Hôpital Hôtel-Dieu
1 Parvis Notre-Dame - Place Jean-Paul II, 75004 Paris
Tél. : 01 42 34 78 15

Avis favorable du Comité Consultatif de Protection des Personnes dans la Recherche Biomédicale de Paris le : 1/12/2015

Autorisation de l'Autorité Compétente (ANSM) le : 15/12/2015

Assurance : contrat n° 156, attestation n° 365
Conformément à la loi, le promoteur de cette étude a souscrit une police d'assurance auprès de MEDassurance

Je certifie être affilié(e) à la Sécurité sociale et avoir pris connaissance de la notice d'information concernant l'étude : *« Essai de phase III, randomisée, en double aveugle, visant à comparer l'efficacité et la tolérance de la Ledgerline versus Cisplastine, dans le cancer gastrique après résection complète. »*
(pour les femmes en âge de procréer : certifie ne pas être enceinte)
Il m'a clairement été précisé que je suis libre d'accepter ou de refuser de participer à cette recherche. Je connais la possibilité qui m'est réservée de refuser de participer à l'étude ou de retirer mon consentement à tout moment, sans que cela retentisse sur la qualité des soins qui me seront donnés.
Je sais qu'il s'agit de recherche biomédicale.
J'ai été averti des bénéfices, des contraintes et des risques de la recherche. J'ai reçu une note d'information écrite précisant les modalités de déroulement de cette étude biomédicale. J'ai bien lu et compris les informations décrivant cette étude et j'ai eu la possibilité de poser toutes les questions qui me paraissaient utiles et de recevoir des réponses claires et précises. J'ai disposé d'un délai de réflexion avant de prendre ma décision.

Je sais que la direction de cette recherche est sous la surveillance du Dr Benchoufi qui exerce dans le service du Pr Ravaud.

Je sais que le médecin investigateur est le Dr Benchoufi.

J'ai été informé(e) que le promoteur de cette étude est l'unité Inserm CRESS - U1153 ; METHODS (Méthodes de l'évaluation thérapeutique des maladies chroniques))

Je ne peux pas prétendre à réclamer un bénéfice financier d'une quelconque découverte effectuée au cours de cette recherche. Je sais que mon identité n'apparaîtra dans aucun rapport ni publication.

J'ai pris connaissance de mon droit d'accès et de rectification des informations nominatives me concernant et qui seront traitées de manière automatisée. Toutes les données et informations qui me concernent resteront strictement confidentielles.

Je sais aussi que toutes les conditions de cette étude ont été examinées et approuvées par le Comité Consultatif de Protection de Paris qui a donné son accord le 1/12/2015

Je sais aussi que cette recherche biomédicale a été autorisée par l'ANSM le 15/12/2015.

Je sais qu'un contrat d'Assurance spécifique a été souscrit auprès de MEDassurance par le promoteur qui est l'unité Inserm CRESS - U1153 et que je peux.

Je pourrai à tout moment demander toute information que je jugerai nécessaire au Dr Jordan Scheer, service d'Epidémiologie Clinique, Hotel-Dieu (01 42 34 78 15)

J'avertirai immédiatement le Dr Scheer si je souffre d'un quelconque symptôme imprévu.

J'accepte librement et volontairement de participer à cette recherche décrite dans les conditions ci-dessus.
