# Supplementary material for: Blockchain protocols in clinical trials: Transparency and traceability of consent
Source: F1000Res. 2018 Feb 1;6:66. Originally published 2017 Jan 23. [Version 5] doi: 10.12688/f1000research.10531.5 (PMC5676196; doi:10.12688/f1000research.10531.5)
Supplement: Supplementary file 1 [file f1000research-6-15062-s0000.tgz › 2ae80474-0dca-4f19-ba4b-5c59d9b49877.zip!/Supplementary File 1/Version 2/Consent form.pdf]

FORMULAIRE DE CONSENTEMENT DESTINE
A LA PERSONNE PARTICIPANT A LA RECHERCHE

Titre de la recherche biomédicale **:** Essai de phase III, randomisée, en double aveugle, visant à comparer l'efficacité et la tolérance de la Ledgerline versus Cisplastine, dans le cancer gastrique après résection complète.

Investigateur coordonnateur :

Mehdi Benchoufi

Coordonnées :
Hôpital Hôtel-Dieu
1 Parvis Notre-Dame - Pl. Jean-Paul II, 75004 Paris
Tél. : 01 42 34 78 15
E-mail : benchoufi.mehdi@gmail.com

Promoteur :

Inserm CRESS - U1153 ; METHODS (Méthodes de l'évaluation thérapeutique des maladies chroniques)

Coordonnées :
Hôpital Hôtel-Dieu
1 Parvis Notre-Dame - Pl. Jean-Paul II, 75004 Paris
Tél. : 01 42 34 78 15

Avis favorable du Comité Consultatif de Protection des Personnes dans la Recherche Biomédicale de Paris le : 1/12/2015

Autorisation de l'Autorité Compétente (ANSM) le : 15/12/2015

Assurance : contrat n° 156, attestation n° 365
Conformément à la loi, le promoteur de cette étude a souscrit une police d'assurance auprès de MEDassurance

Je soussigné(e)
Nom :
Prénom :
Date de naissance :
Adresse :
certifie être affilié(e) à la Sécurité sociale et avoir pris connaissance de la notice d'information concernant l'étude : *« Essai de phase III, randomisée, en double aveugle, visant à comparer l'efficacité et la tolérance de la Ledgerline versus Cisplastine, dans le cancer gastrique après résection complète. »*
(pour les femmes en âge de procréer : certifie ne pas être enceinte)
Il m'a clairement été précisé que je suis libre d'accepter ou de refuser de participer à cette recherche. Je connais la possibilité qui m'est réservée de refuser de participer à l'étude ou de retirer mon consentement à tout moment, sans que cela retentisse sur la qualité des soins qui me seront donnés.
Je sais qu'il s'agit de recherche biomédicale.
J'ai été averti des bénéfices, des contraintes et des risques de la recherche. J'ai reçu une note d'information écrite précisant les modalités de déroulement de cette étude biomédicale. J'ai bien lu et compris les informations décrivant cette étude et j'ai eu la possibilité de poser toutes les questions qui me paraissaient utiles et de recevoir des réponses claires et

précises. J'ai disposé d'un délai de réflexion avant de prendre ma décision.

Je sais que la direction de cette recherche est sous la surveillance du Docteur Benchoufi qui exerce dans le service du Pr Ravaud.

Je sais que le médecin investigateur est le Docteur Benchoufi.

J'ai été informé(e) que le promoteur de cette étude est l'unité Inserm CRESS - U1153 ; METHODS (Méthodes de l'évaluation thérapeutique des maladies chroniques))

Je ne peux pas prétendre à réclamer un bénéfice financier d'une quelconque découverte effectuée au cours de cette recherche. Je sais que mon identité n'apparaîtra dans aucun rapport ni publication.

J'ai pris connaissance de mon droit d'accès et de rectification des informations nominatives me concernant et qui seront traitées de manière automatisée. Toutes les données et informations qui me concernent resteront strictement confidentielles.

Je sais aussi que toutes les conditions de cette étude ont été examinées et approuvées par le Comité Consultatif de Protection de Paris qui a donné son accord le 15/06/2016

Je sais aussi que cette recherche biomédicale a été autorisée par l'ANSM le 01/07/2016.

Je sais qu'un contrat d'Assurance spécifique a été souscrit auprès de MEDassurance par le promoteur qui est l'unité Inserm CRESS - U1153 et que je peux.

Je pourrai à tout moment demander toute information que je jugerai nécessaire au Docteur Scheer, service d'Epidémiologie Clinique, Hotel-Dieu (01-42-34-78-15)

J'avertirai immédiatement le Docteur Scheer si je souffre d'un quelconque symptôme imprévu.

J'accepte librement et volontairement de participer à cette recherche décrite dans les conditions ci-dessus.

Fait à ...., le..........................

Nom et signature de la personne participante
(*ou en cas d'impossibilité, attestation d'un tiers, personne indépendante de l'investigateur et du promoteur (Art. L 1122-1-1)) (lien)*

Fait à .... le...........................

Nom et signature de l'investigateur
