# Supplementary material for: Blockchain protocols in clinical trials: Transparency and traceability of consent
Source: F1000Res. 2018 Feb 1;6:66. Originally published 2017 Jan 23. [Version 5] doi: 10.12688/f1000research.10531.5 (PMC5676196; doi:10.12688/f1000research.10531.5)
Supplement: Supplementary file 2 [file f1000research-6-15062-s0001.tgz › 895c29be-d132-41f6-b65a-aa88ff5155fd.zip!/Supplementary File 2/Version 0/Consent form.pdf]

CONSENT FORM TO THE PERSON PARTICIPATING IN RESEARCH

Title of the biomedical research: Phase III, randomized, double-blind trial to compare the efficacy and tolerance of Ledgerline versus Cisplastine in gastric cancer after complete resection.

Coordinating Investigator: Dr. Mehdi Benchoufi
Contact information :
Hôpital Hôtel Dieu
1 Parvis Notre Dame Pl. Jean-Paul II, 75004 Paris Tel: 01 42 34 78 15
Email: mehdi.benchoufi@aphp.fr

Sponsor:
Inserm CRESS U1153; METHODS (Methods of therapeutic evaluation of chronic diseases)
Contact information :
Hôpital Hôtel Dieu
1 Parvis Notre Dame Place Jean-Paul II, 75004 Paris Tel: 01 42 34 78 15

Favorable opinion of the IRB on 15/06/2016

Authorization of the Competent Authority (ANSM) on: 01/07/2016

Insurance: contract n ° 156, attestation n ° 365
In accordance with the law, the sponsor of this study has taken out an insurance policy from MEDassurance

I certify

Your Name:

First name:

Birth date :

Address :

that I am a Social Security Affiliate and have read the information sheet for the study: "Phase III, randomized, double-blind trial to compare the effectiveness and tolerability of the Ledgerline Versus Cisplastine, in gastric cancer after complete resection. "
(for women of childbearing age: certifies not to be pregnant)

It was clear to me that I am free to accept or refuse to participate in this research. I am aware of the possibility of refusing to participate in the study or withdrawing my consent at any time, without affecting the quality of the care I will receive.
I know it's biomedical research.

I have been warned about the benefits, constraints and risks of research. I received a written briefing note outlining how this biomedical study will be conducted. I have read and understood the information describing this study and have had the opportunity to ask any questions that appeared to me useful and to receive clear and precise answers. I had a period of reflection before I made my decision.

I know that the direction of this research is under the supervision of Dr. Benchoufi who practices in the service of Professor Ravaud.

I know the investigating doctor is Dr. Benchoufi.

I have been advised that the sponsor of this study is Inserm CRESS U1153 unit; METHODS (Methods of therapeutic evaluation of chronic diseases)

I can not claim a financial benefit from any discovery made during this research. I know that my identity will not appear in any report or publication.

I have read my right to access and to rectify personal informations that concern myself and that will be processed in an automated manner. All data and information concerning me will remain strictly confidential.

I also know that all the conditions of this study were examined and approved by the IRB of Paris which gave its agreement on 15/06/2016
I also know that this biomedical research was authorized by ANSM on 01/07/2016.

I know that a specific insurance contract has been signed with MEDASSurance by the sponsor that is Inserm CRESS U1153 unit.

I may at any time request any information I deem necessary to Dr. Jordan Scheer, Department of Clinical Epidemiology, Hôpital Hôtel Dieu (Tel: 01 42 34 78 15)
I will immediately let Dr. Sheer know if I have any unforeseen symptoms.

I accept freely and voluntarily to participate in the research described in the conditions above.

Done at ...., on ....

Name and signature of the participant

(Or in case of impossibility, attestation of a third person, person independent of the investigator and the sponsor)

Done at .... on ....

Name and signature of investigator
