# Supplementary material for: Blockchain protocols in clinical trials: Transparency and traceability of consent
Source: F1000Res. 2018 Feb 1;6:66. Originally published 2017 Jan 23. [Version 5] doi: 10.12688/f1000research.10531.5 (PMC5676196; doi:10.12688/f1000research.10531.5)
Supplement: Supplementary file 2 [file f1000research-6-15062-s0001.tgz › 895c29be-d132-41f6-b65a-aa88ff5155fd.zip!/Supplementary File 2/Version 1/Protocol.pdf]

INFORMATION NOTE TO PATIENT PARTICIPATING IN RESEARCH
Updated Version on 18 July 16

Title of the biomedical research: Phase III, randomized, double-blind trial to compare the efficacy and tolerance of Ledgerline versus Cisplastine in gastric cancer after complete resection.

Coordinating Investigator: Dr. Mehdi Benchoufi

Contact information:
Hôpital Hôtel Dieu
1 Parvis Notre Dame Pl. Jean-Paul II, 75004 Paris
Tel: 01 42 34 78 15
Email: mehdi.benchoufi@aphp.fr

Sponsor:
Inserm CRESS U1153; METHODS (Methods of therapeutic evaluation of chronic diseases)
Contact information :
Hôpital Hôtel Dieu
1 Parvis Notre Dame Pl. Jean-Paul II, 75004 Paris
Tel: 01 42 34 78 15

Favorable opinion of the IRB on 15/06/2016

Authorization of the Competent Authority (ANSM) on: 01/07/2016

Insurance: contract n ° 156, attestation n ° 365

In accordance with the law, the sponsor of this study has taken out an insurance policy with MEDassurance.

Dear Madam, Dear Sir,

We inform you that a recent study shows that ledgerline can be responsible for rare oesophageal ulcerations and exceptional myalgia or uveitis. In addition, osteo-necroses of the mandible were described when the drug is administered intravenously at high doses.

To the extent of this new information, we present to you all the information for which we ask for a renewed consent.
We leave you a few days to reflect on this proposal and remain at your disposal to answer all your questions.

* **Background:**
Cisplastine, is the reference chemotherapy following a complete resection in gastric cancer. Previous studies seem to suggest that Ledgerline would be more effective than Cisplastine.

* **Purpose of the research:**
To evaluate the efficacy of Ledgerline on relapse-free survival in patients with gastric cancer after complete resection. Assess the toxicity of this drug.

* **Methodology:**
Patients included in this study are patients with stomach cancer who have undergone complete resection.
Following a random draw, patients will be divided into 2 groups: one group receiving Cisplastine, one group receiving Ledgerline (randomization).
Neither the patient nor the physician will be aware of the group in which the patient was assigned (double blind).

The duration of the follow-up will be 4 years. Surveillance will be done every 6 months, including a thoraco-abdomino-pelvic CT scan, and a clinical examination.

If during the study statistical analyzes seem to suggest that one of the treatments is more effective than the other, the study will be suspended and patients will benefit from the treatment that appears to be the most effective.

* **Expected benefits:**
Improved survival without relapse.

* **Constraints, obligations and restrictions imposed, discomfort, foreseeable risks:**
Patients will receive two a week for 12 weeks one of 2 molecules, in one of the oncology departments participating in this study.
Cisplastine and Ledgerline are two molecules that can cause significant vomiting. Antiemetic treatment will be prescribed to the patient in this case.
Cisplastine and Ledgerline may cause hair loss and neutropenia.

* **Side effects:**
We inform you that a recent study shows that Ledgerline can be responsible for rare oesophageal ulcerations and exceptional myalgia or uveitis. In addition, osteonecrosis of the mandible has been described when the product administered intravenously at high doses.

* **Medical fees:**
Your collaboration in this biomedical research protocol will not involve financial participation on your part. In accordance with the law, all costs related to the study will be borne by the study sponsor.

* **Confidentiality:**
Any information about you collected during this trial will be treated confidentially.
Only those responsible for the study and the health authorities may have access to these data. With the exception of these persons (who will treat the information in the strictest respect of medical secrecy), your anonymity will be preserved. The results of the study will not be published individually.
The data recorded through this study will be subject to a computerized treatment by the promoter.
In the case of personal data, you are entitled at all times to the right of access and rectification of the data concerning you to the persons responsible for the study and, with regard to information of a medical nature, this right is exercised by Dr Benchoufi in accordance with Law 78-17 of 6 January 1978 related to Information Technology, Files and Liberties, as amended by Law 94-548 of 1 July 1994, related to the processing of personal data for the purpose of Research in the field of health. This project received a favorable opinion from the National Commission for Information Technology and Civil Liberties on 15 June 2016.

* **Modalities of transmission of the overall results of the research:**
In accordance with article L 1122-1 of the Public Health Code (March 2002 law on patients' rights), the overall results of the study can be communicated to you if you wish.

You will be informed of the results of this study as soon as they become available.

This project is funded by APHP and in partnership with the National Cancer Institute (INCa).
We leave you a few days to reflect on this proposal and remain at your disposal to answer all your questions.

After reading this information note, do not hesitate to ask any questions you may have to your treating doctor. This information note must be retained.

We thank you for your cooperation and, if you agree, ask you to sign the consent form.

Done at ...., on ....

Name and signature of the participant

(Or in case of impossibility, attestation of a third person, person independent of the investigator and the sponsor)

Done at .... on ....

Name and signature of investigator
