# Supplementary material for: Blockchain protocols in clinical trials: Transparency and traceability of consent
Source: F1000Res. 2018 Feb 1;6:66. Originally published 2017 Jan 23. [Version 5] doi: 10.12688/f1000research.10531.5 (PMC5676196; doi:10.12688/f1000research.10531.5)
Supplement: Peer review history

# Peer review: Referee response for version 4

**DOI:** 10.5256/f1000research.14293.r28834

**Published:** 2018-01-08

**Authors:** Suveen Angraal

**Affiliations:** 1 Center for Outcomes Research and Evaluation, Yale New Haven Hospital (YNHH), New Haven, CT, USA

In the current version, the authors have made improvements to the manuscript from the previous version; the idea entails the use of blockchain in the consent processing in a clinical trial. However, I still have some pending concerns which, if authors agree to work on, can further the manuscript’s message to readers.

 In the first report, I pointed to identity verification, where the consent can be verified but the patient him/herself cannot be verified. Although the authors have explained how the keys can be stored in the browser itself for verification. This approach has a lot caveats in implementation. I would encourage the authors to address this as a limitation, rather than delving further into the incomplete annotations. Secondly, authors address that even if malicious investigator consented in behalf of the subject, this very subject will have to show up for the participation. This indeed is true, but doesn’t address the falsification of the data.

 With last few months of 2017, price of bitcoin token has skyrocketed. Given that authors have used bitcoin blockchain, how do authors envision the cost of transactions when consenting, with changing value of bitcoin token? Would a private blockchain be better suited for actual implementation in a clinical trial? Furthermore, if a private blockchain is used, is sybil attack more probable?

 I had previously raised the importance of explaining the meaning of ‘public’ in a blockchain and what data will be available ‘publicly’. Although authors have explained this in the response letter, they can still add a few lines about chainscript and its role in transparency.  Further, authors state that “implementing a ‘privacy by design’ technology, and archiving securely and transparently any dataset that needs to be stored, is a game changer toward improving enrolment phase methodology”. First, authors overstate the implication of how archiving securely is a game changer. Secondly, a secure and transparent archiving can be achieved without blockchain as well. The manuscript will improve further if authors circumspect such statements. Additionally, statements like “we evoked a possible improvement in the enrolment rate in clinical trials by empowering patients and granting them information and control over the enrolment phase” seems overselling the underlying message.

 The patient empowerment is still not clear enough in the manuscript. In the current logistics of a clinical trial where consent is taken on the paper, the patients are empowered to control their participation in the enrolment phase, and see who has the access to their data (which includes consent). It would further improve the manuscript if authors highlight what additional benefit blockchain brings in empowering the patients. Otherwise, I would encourage authors to refrain from making such assertions.

 The reference for Namecoin just directs to the Wikipedia homepage (https:// en.wikipedia.org/wiki). The authors should careful when citing the references.

I have read this submission. I believe that I have an appropriate level of expertise to confirm that it is of an acceptable scientific standard, however I have significant reservations, as outlined above.

# Response

**Published:** 2018-01-25

**Authors:** mehdi benchoufi

**Affiliations:** Hotel-Dieu Hospital, Paris Descartes University, France

Dear reviewer,

Thank you for giving us the opportunity to make the text more clear and by so improving its overall quality

**“In the first report, I pointed to identity verification, where the consent can be verified but the patient him/herself cannot be verified. Although the authors have explained how the keys can be stored in the browser itself for verification. This approach has a lot caveats in implementation. I would encourage the authors to address this as a limitation, rather than delving further into the incomplete annotations.”**

In our last answer where we asked to the “physical identity crisis”, we stressed the fact that a decentralized way to manage the key is not a solution to ascertaining the identity of the user. However, we indicated that there are ways to bind public key generation to an email, consisting in attaching some token in the email sent to the patient so that the public key generated can hold this information and so that the stakeholders can infer to which email a public key is bound.

But of course, email is not a strong and satisfactory solution. A good solution should be based on providing patients with material devices such as USB device to hold the keys and ideally to implement some KYC solutions modeled on the bank sector standards for instance. In the manuscript, we indicated in "In a production application (…) implementing a Know Your Customer (KYC)-like solution" that we should rely on KYC standard solution rather than binding public key with email, which is a quite poor way to ensure identity.

So, we updated the manuscript, synthesized briefly the part related to the process of binding key pairs and email, deleted the mention of the Namecoin blockchain which can be misleading, and stress the fact that binding email and key pair generation in some way does not provide some consistent way to tackle the identity issue.

**Secondly, authors address that even if malicious investigator consented in behalf of the subject, this very subject will have to show up for the participation. This indeed is true, but doesn’t address the falsification of the data.**

Thank you for this remark. If indeed a key forgery happened and that a malicious investigator consents in behalf of a subject, then indeed a patient won’t show up and the data would be definitely falsified. According to the incorruptible nature of the data stored in the Blockchain, there is no way back, and we are going to make this point clearer in the manuscript. However, to monitor the eventuality of data falsification, we can send back, whether a patient consented or not, an email to him with this very information, which is a supplementary lock in the process and which was indeed done in our POC. (This lock could be even improved if the email was sent after a Smart Contract has been hit by a transaction happening when the user chooses consenting or not). If some user then complains, we can ignore the related Blockchain transaction.

Thereafter, we should underline that data invention do not fall into the scope of misconducts directly avoidable with Blockchain, even it seems to be harder than frauding manually: creating pair of keys, hacking around the timestamping of every event, hiding the IP of web requests.

From a more general point of view, we are aware of the limitation the reviewer emphasizes and in the actual state of this implementation, our main hypothesis here is that there are stakeholders who have the admin-permissions to create key pairs are to be trusted. By the current times, clinical trials heavily relies on the existence of such stakeholders such as investigators, sponsors, regulatory agencies, FDA or EMA or IRBs. So there is some trust to attach to the stakeholder that we trust the most, and devote to him the generation of pair keys. For example, if there is a mistrust towards the investigator, then keys could be generated by the sponsor or any other external party under the supervision of the sponsor, a regulatory agency or the IRB. The advantage of our current solution is that it can be directly implemented for clinical trials in the way they are conducted today.

In any case, the development of our approach should lead us quickly to implement key management on the user side to control this kind of forgery from a malicious investigator. We updated the text to mention this issue and to stress the fact that there is indeed no way to undo a falsified data.

*Remark:*

We should mention that KYC adapted in the context of Blockchain is an active area of development. Many startups and even public institutions begin to propose some solutions enabling consensus-driven identity for personal entities, helping avoid redundancy and high costs of traditional KYC processes. Companies such as KYC-Chain (1) or Norbloc (2) provide proof-of-identity solutions, recently Singapour government, with regards to the Anti-Money Laundering (AML) requirements and considering the costs of the KYC usual implementation in the finance industry, proposed a POC which is announced as the most advanced one in this field  to companies (3). We are regularly watching to advances in this field since it will help for us designing better and flexible solutions where user control, manage their digital identity and most importantly would allow a generic approach of KYC, reusable from one platform to an other, and so could enable flexible usage while guaranteeing security and sincerity of the process.  

(1) https://kyc-chain.com/

(2) https://norbloc.com/

(3) https://cointelegraph.com/news/first-blockchain-kyc-prototype-hits-singapore-banking-sphere/

**With last few months of 2017, price of bitcoin token has skyrocketed. Given that authors have used bitcoin blockchain, how do authors envision the cost of transactions when consenting, with changing value of bitcoin token? Would a private blockchain be better suited for actual implementation in a clinical trial? Furthermore, if a private blockchain is used, is sybil attack more probable?**

Indeed the price of the bitcoin currency has grown rapidly, passing from around 1000$ at the beginning of 2017 to 15.000€ at the moment of the writing (09.01.18). Fluctuations of the currency is quite a structural fact that led developers to worry about designing transactions, when using Bitcoin as an API (Application Program Interface), that can be as resilient as possible to these fluctuations. That’s the reason why we took care to make use of a data-structure that can absorb by design the volatility of the currency rate. However, even if we have a large flexibility to group transactions and so validate an important amount of data in one single transactions and even if we have room to maneuver by playing on the lever of raising the number of data we attach to the data-structure for a single transaction balanced by the longer delay of triggering a transaction, we have some dependency to the Bitcoin currency through the fees we have to pay for a transaction. We can group up to several ten thousands of proof-of-data in a single transaction and the fee we would pay in that case would be around 20$ (at the moment of the writing 22.01.18). We will report this issue in the manuscript.

We won’t go into too much technical details, but the following matters to get an understanding of the level of resilience, although still sensible, of the data-structure we used to the fluctuating evolution of the Bitcoin currency.

Indeed, we choose to use a Merckle tree to hold the data from which we want to memorize a proof-of-existence. As explained in our previous responses, the data are stored in a binary tree, and successively hashed by pair of leaves by pair of leaves. More precisely, the integrity of the data-structure relies on the hashing of pair of leaves nodes which outputs the hash of the parent node of these very leaves. This process bubbles up until the root of the Mercke tree is reached. This root stores a hash and the point is that we only need to store in the Blockchain the hash of the so-called Merckle root. Technically, this piece of data is put in a so-called OP_RETURN field that is now widely used as a hack for storing lightweight data into the blockchain. Now, when we want to prove the existence of a set of data, we have to compute their hash again pair of leaves by pair of leaves up to the root and compare this one against the OP_RETURN hash. In case the hashes are identical then we conclude the integrity of the data we want to check. If in the tree, one single unity of data is corrupted then the entire data-structure breaks down and we can conclude that these were corrupted.

Now, when grouping transactions by batch, we can store proof of huge amount of data. Indeed, we can group transaction by slices of 1000, 10.000 or even more batches of data, each hash corresponding to a possibly large document. This way, we can adapt to the high variance behaviour of the Bitcoin currency by raising up the number of transactions we want to throw in the Blockchain, we could even multiply by an other factor of 10 the number of batches of hashes of data to reach 100.000, but we would have to wait to get this volume of data before triggering the Blockchain transaction and be pejorated by the delay between the data we get and the data of which we store the proof.

Besides, with regard to the remark about private Blockchain and sensitivity to a Sibyl attack, we would argue somehow the contrary, private Blockchain in that they may control the identity of who is allowed to participate to the network, makes it quite unlikely for an attacker flow the network with one identity and act as a multiple one detaining multiple keys, and so sybil attack the system. On the other side, public Blockchain is not totally damp-proof to Sybil Attack though it is made very difficult, because of the proof-of-work being increasingly difficult when the number of nodes the attacker creates and hides behind increases (1), and because of the randomness of outbound connections of a user connecting to others. However, if private Blockchain may grant a certain level of control on the process occuring in the network, we think, as mentioned above in one of our responses, that private Blockchain may be a way to re-centralize the network and take less advantage of distributed the trust in an open network. The choice of this kind of structure can be relevant in some situation but needs strong motivations and understanding of the technological background when one gives certain of its advantages to the Blockchain.

We updated the manuscript to make this point clearer and say of few words about the evolution of the currency that was not that volatile the first time we submitted to the F1000 review.

**Remark 1**: There are lots of debate to tackle the issue of a better control of the Bitcoin fees, some developers advocate the solution of a bigger size of Block. This gave rise to a forked Bitcoin Blockchain, called Bitcoin Cash, which leveled up to 8mb the data that can be fit to a block, versus 1mb in the core Bitcoin implementation.  

**Remark 2**: Bitcoin core developers are testing an off-chain payment channel system called “Lightning” and is supposed to increase the number of transactions per unit of time and to decrease the transaction fees.

**Remark 3**: There are current Blockchain implementation based on indexing crypto-currency value to other assets, such as dollars. Stabl is such as an example with the promise to give more stability to the currency value.

(1) https://arxiv.org/pdf/1706.00916.pdf

(2) https://hyperledger.org/

(3) https://blog.variabl.io/stabl-bringing-stable-tokens-and-derivative-products-to-the-ethereum-blockchain-df4d5eba89d9

**I had previously raised the importance of explaining the meaning of ‘public’ in a blockchain and what data will be available ‘publicly’. Although authors have explained this in the response letter, they can still add a few lines about chainscript and its role in transparency.**

In our previous response, we explained what we meant by “publicly verifiable”. To summarize, the interactions that happens throughout the consent process are tracked on the Blockchain, restituted in a Chainscript document, the hashes of which can be checked against the Bitcoin Blockchain either manually or public website offering such services. The ChainScript document can be described as a JSON standard for proof-of-process (1). It is a standard to expose all the steps of the verifiable process one want to design and that leads to the Blockchain transactions. The different steps are attached meta-data and these meta-data are organized with respect to the historicity of the sequence of transactions that were grouped, hashed and then stored in the Blockchain.

Technically, the process is broken down into steps, the process corresponds to a so-called Chain Map and a step correspond to a Chain Segment, each of which holds a reference to a previous Chain Segment which stands for the (n-1) step. The ChainScript is built up all along the history of the process with the help of so-called Chainscript Agents, which are responsible to execute scripts enabling “state transitions” (in the computer language of state machines), corresponding to move from one step to an other one in the process we want to check.  

This way, the Chainscript is a full, transparent, checkable history of the life of the data we stored in the Merckle tree. ChainScript is an open standard.  

We updated the manuscript to precise this point and explain how the Chainscript improves transparency in reporting point by point and through an homogeneous document the event history of a process.

(1) http://chainscript.io/

**Further, authors state that “implementing a ‘privacy by design’ technology, and archiving securely and transparently any dataset that needs to be stored, is a game changer toward improving enrolment phase methodology”.**

**First, authors overstate the implication of how archiving securely is a game changer. Secondly, a secure and transparent archiving can be achieved without blockchain as well. The manuscript will improve further if authors circumspect such statements.**

When we enunciate that archiving functionality is a game changer, we had in mind all the issues related to the consent phase main of which are: failure to obtain written informed consent, failure to provide copies of the document to subjects, consent document not signed or not dated, missing pages in consent document provided to subjects, failure to re-consent when new information becomes available, use of wrong forms, use of expired, non-validated or unapproved forms, the only document last page kept in study files. We see from there that implementing a technical pipeline to connect consent process events and Blockchain in order to record consistently timestamped facts helps tackle these issues. The concern about consent process is regularly raised by the FDA, and in the manuscript we referred to articles that treats of this matter.

However, we updated our manuscript to propose a softer expression and precise that the archiving is an incorruptible history-oriented timestamped process.

Besides, thank you for pointing out this problematics and indeed archiving can be done without Blockchain. Before Blockchain was built, computer science researchers and game theorists conceived some ways to achieve security and transparency, especially DLT, Distributed Ledger Technologies (DLT), this allowed archiving consistently data and of which Blockchain is an improvement, but in these systems there are no “proof-of-work”, i.e. validation by random users who proves in some way, by providing CPU intensive power in the case of Bitcoin Blockchain, that they are substantially engaged in the transaction validation. Here, when we claim for a better security and transparency, we allude to the distributed nature of the protocol and critically to its openness, everyone being part of the proof-of-work system, no authentication to join the network being required (we mention that there are of course some recent proof-of-stake or other processes to make network participants engaged in the transaction validation which do not consist in providing CPU power).

So, Blockchain is a community consensus driven technology.

The “proof-of-work” system integrates the community of peers in the validation process. It happens to say Blockchain supports a trustless system, in fact we could say more appropriately that each node if the network detains part of the trust. So, in this way, we estimate Blockchain is more robust, secure, in the sense that a distributed network circumvents the issue of “single node point of failure”, and transparent. The wide adoption of Bitcoin, Ethereum or other community consensus driven technologies, is related to their open or so to say the so-called “permissionless” nature, which ensures that communities of peer users are not selected a priori by any central authority so that the validation of transactions through an open and inclusive proof-of-work process is robust.

On a more broader view, what is commonly said about the core specificity with regards to the attributes that can be appended to the Bitcoin Blockchain technology, is that this distributed database helps achieve in the same time three goals that were not met altogether by precedent technologies: consensus about rules, consensus about history, consensus that coins are valuable. With this strong consensus guardrails, we think that our choice of public blockchain, here the Bitcoin network - but we could have made the choice of ethereum -  helps us reach a satisfactory level of transparency and security.   

**Remark**: DLT technologies, among which we rank private Blockchain, are more prone to corruption of the informations if inclusion gatekeeping to the network led to go to an illicit agreement.

Of course, we don’t claim that Bitcoin’s Blockchain is the absolute standard but it brings quite good guarantees for security and transparency since corruption of the network would suppose a majority of the nodes - this is the famous 51% attack which is yet a theoretical attack at least on the Bitcoin network - and there are currently great efforts to improve these technologies. A recent technology hitting the headlines is HashGraph, it especially provides an improvement, not on the security and transparency side, but on the matter of “proof-of-work” which requires a lot of computing power, so that more transactions could happen per unit of time.

We updated the document  we might indicate that there are other ways to achieve some level of security and transparency in archiving data, though Blockchain gives a substantial improvement.

**Additionally, statements like “we evoked a possible improvement in the enrolment rate in clinical trials by empowering patients and granting them information and control over the enrolment phase” seems overselling the underlying message. The patient empowerment is still not clear enough in the manuscript. In the current logistics of a clinical trial where consent is taken on the paper, the patients are empowered to control their participation in the enrolment phase, and see who has the access to their data (which includes consent). It would further improve the manuscript if authors highlight what additional benefit blockchain brings in empowering the patients. Otherwise, I would encourage authors to refrain from making such assertions.**

Indeed our formulation is not clear and can be misleading in some sense.

But let us give some elements that led to us to choose this formulation. Blockchain leads to a community driven consensus to validate proof-of-events whose digital representation is encoded as a block in the public ledger. The level of improvement we envision is not at the level of control of the participation itself but rather at the level of trust in the consent process as a whole, which is an important issue with regards to ongoing debates on patients rights, privacy at the time of mistrust in public authorities or private institutions, especially in the pharmaceutical area.

Since Blockchain brings together elements of technology that garantee a strong level of consistency of the process it monitores, and given that each user of the network detains a piece of trust, we think that patients can be more confident in the consent process as a whole and have a supplementary level of control on the integrity and the lifetime of the data whose proof is stored in the Blockchain.   

However, since in the current implementation, the user are not in a situation to manage directly their keys, the level of control on the Blockchained consent collection steps is not plainly satisfactory and so user empowerment that we claim has to be improved and we updated the manuscript in order to take into account your remark.

**The reference for Namecoin just directs to the Wikipedia homepage (https:// en.wikipedia.org/wiki). The authors should careful when citing the references.**

Thank you for your vigilance, we deleted this reference when taking into account one of your preceding remarks.

# Peer review: Referee response for version 3

**DOI:** 10.5256/f1000research.12989.r26847

**Published:** 2017-11-14

**Authors:** Timothy Nugent

**Affiliations:** 1 Corporate Research and Development, Thomson Reuters, London, UK

This article describes a proof of concept system which leverages the Bitcoin blockchain and Chainscript proof of process in order to enforce patients' informed consent during clinical trials. The method relies on the timestamping characteristic of blockchain transactions to ensure that consent was given by the patient in light of modifications to the trial protocol.

 While it is possible to follow the description of the method, there are many typographic and grammatical errors. The manuscript needs to be improved and these numerous errors corrected.

 There are a number of issues that need to be addressed.

- Bitcoin here seems inappropriate for the task at hand. The authors discuss Ethereum but choose Bitcoin as it is perceived to be more stable and immutable. Both chains have experienced hard forks, and both are susceptible to 51% attacks. Ethereum's flexible smart contracts would seem entirely appropriate to use for data storage here, given that any tampering of the chain would be much more likely to seek economic gain than attempt to modify the trial record. In any case, even on Ethereum the cost of such an attack is essentially prohibitive.
- If using a public chain of any sort, the cost in terms of transactions fees and confirmation times should be discussed. For large scale trials, these costs are likely to be substantial.
- Authors state that subjects are assigned a key - assigned by who? Surely this introduces a security risk as there exists no mechanism to ensure that these keys are deleted by whoever creates and assigns them. There are various mechanism that allow key pairs to be created by end users (e.g. in a web browser using client-side Javascript)
- How are public keys associated with subject identities (or their email addresses)? Linking these is non-trivial (assuming the keys are not generated by a central entity). Services exists to link e.g. OAuth 2 tokens with blockchain addresses - the manuscript should at the very least cover some of the identity solutions that may be appropriate here.

I have read this submission. I believe that I have an appropriate level of expertise to state that I do not consider it to be of an acceptable scientific standard, for reasons outlined above.

# Response

**Published:** 2017-12-08

**Authors:** mehdi benchoufi

**Affiliations:** Hotel-Dieu Hospital, Paris Descartes University, France

Dear Reviewer, 

We have just answered to the last two referees report. We are going now to answer yours.

Thank you for your understanding and patience

Best regards,

# Peer review: Referee response for version 3

**DOI:** 10.5256/f1000research.12989.r26848

**Published:** 2017-11-06

**Authors:** Suveen Angraal, Wade Schulz

**Affiliations:** 1 Center for Outcomes Research and Evaluation, Yale New Haven Hospital (YNHH), New Haven, CT, USA; 2 Center for Outcomes Research and Evaluation, Yale New Haven Hospital (YNHH), New Haven, CT, USA; 3 Department of Laboratory Medicine, Yale School of Medicine, New Haven, CT, USA

In this proof of concept (POC), the authors have implemented the use of blockchain technology to obtain secure, unfalsifiable consent in a clinical trial. Although the authors have a done a good job in explaining the methods of this POC and how they achieved the aims of the study, there are still remain some concerns, which, if addressed, can substantially improve the manuscript.

 While talking about a public ledger, authors should explain what ‘publically verifiable’ means in the context of a clinical trial. To the understanding from the manuscript, the authors have used the timestamping feature of blockchain as the basis to explain how the verification will work. This is a vital facet of blockchain, but the extent of this in a clinical trial consent would only be limited to the fact that a consent was signed at a particular time. Whether the consent can be ‘verified’ to be legitimate is still unclear. This then boils down to the physical identity crisis, which in itself can be a separate research manuscript. The authors can add a paragraph explaining the limitations of this crucial drawback in detail. So far there is only a brief description in the manuscript. Additionally, a public ledger may be unpopular with patients for enrollment. A detailed explanation on what ‘public’ means and which entities will be able to see the participant’s consent data from the distributed ledger needs to be highlighted.

 In the introduction, the authors have given examples where the consents have been mishandled. Some of the examples are not specific to the consent mishandling, for example, the DECREASE studies where the concern was of falsifying the data as a whole. The manuscript would look more precise if examples of clinical trials or research studies specific to consent mishandling are given. Secondly, the authors highlight the empowering the patients and improved enrollment in a clinical trial. It would help if authors expand on patient empowering. From the manuscript’s current structure, the patient empowerment remains at same status as it would be in a written consent, to the extent that patients can pull out of the study whenever they want and can see which entities are involved in the study. Hence, a discussion about this crucial aspect is worth including in the manuscript. Thirdly, the general descriptions of blockchain (ie, 'a giant public datastore') are not entirely correct, and the strengths of the technology ('backbone of the circulation of digital assets, powering any kind of services…') are overstated. Furthermore, the unfalsifiable nature of any particular blockchain depends on a distributed deployment, as in many implementations, the person/group owning a majority of the miners could change or alter data. Authors should elaborate on these specifics so that the readers have the understanding of the specific nuances of blockchain. Finally, many of the concerns and issues with consent noted in the introduction (not obtaining IRB approval of new consent forms, etc) are administrative issues – auditing of these updates could be done with the system presented here, but use of blockchain alone would not necessarily prevent all of these issues from occurring.

 In the methods section, the authors argue on why they opted for bitcoin blockchain (because of stability, immutability, large mining network). Given that the authors rely on smart contracts for future developments, it is difficult to understand why they did not opt for the Ethereum which has the same benefits as those of bitcoin blockchain with added benefit of smart contract platform. The manuscript would improve if the authors give more context on their choice, and also, what they envision would be used in a real-world application of their POC in a clinical trial.

 The authors state in prior reviews that the exact chaincode/blockchain data cannot be shared for review purposes due to the presence of private information. While this is undoubtedly true, it is difficult to understand, based on the current description, how the authors envision deploying a decentralized system if this is an issue. We agree with concerns from other reviewers regarding how much of a proof of concept was implemented, and concerns with key steps of the process such as identify verification. While it is understood that this is a proof of concept, it would be helpful as a method paper to at least detail how these issues would be resolved in an actual application deployed beyond the proof of concept stage.

 Another aspect of the blockchain important to discuss is the cost effectiveness. Blockchain is resource intensive technology which requires a lot of computational power. Given that the funding of the many research studies is limited, a calculated investment is always required. If authors can talk a little about the logistics and cost effectiveness of this blockchain setup, it would bring a new dimension to the manuscript.  

 Finally, the manuscript can improve on the language. Even after three revisions, there are typing and grammatical errors which are concerning. Manuscript can improve a lot if the authors can thoroughly proof read the manuscript and revise accordingly.

We have read this submission. We believe that we have an appropriate level of expertise to confirm that it is of an acceptable scientific standard, however we have significant reservations, as outlined above.

# Response

**Published:** 2017-12-06

**Authors:** mehdi benchoufi

**Affiliations:** Hotel-Dieu Hospital, Paris Descartes University, France

Dear reviewer, 

Thank you for giving us the opportunity to make the text more clear and by so improving its quality

**In this proof of concept (POC), the authors have implemented the use of blockchain technology to obtain secure, unfalsifiable consent in a clinical trial. Although the authors have a done a good job in explaining the methods of this POC and how they achieved the aims of the study, there are still remain some concerns, which, if addressed, can substantially improve the manuscript.**

**While talking about a public ledger, authors should explain what ‘publically verifiable’ means in the context of a clinical trial. To the understanding from the manuscript, the authors have used the timestamping feature of blockchain as the basis to explain how the verification will work. This is a vital facet of blockchain, but the extent of this in a clinical trial consent would only be limited to the fact that a consent was signed at a particular time. Whether the consent can be ‘verified’ to be legitimate is still unclear.**

Here, we explain what we mean by “Publically verifiable”. The main output of the experience is what we called a ChainScript document. This document summarize all the tracked interactions of the user with our web platform: for instance, consent status, protocol versions, re-consent status with regards to a specific version. For each user participating to the POC and each tracked website actions, a transaction was triggered into the bitcoin network.  When the bitcoin network validates a transaction then a block is added to the ongoing blockchain. Our ChainScript documents stands for this block. Since, bitcoin is a public blockchain, anyone can verifies that a transaction is indeed present in some block of the blockchain. In our context, suppose you want to verify that the consent status for some user, then one have to take the corresponding hash of the alleged transaction in the ChainsScript document and verify that this really exists in the blockchain. So now, how to verify that this transaction exists in the blockchain : either verifies `by hand`, one downloads the whole bitcoin blockchain, which corresponds to around 160Gb [1] (at the time of the writing), and check the actual transaction exists. In a more useful way, one use some services offered by public website that verifies that the transaction one is looking to verify actually exists and check the proof-of-data is indeed in this transaction. `blockchain.info` or `live.blockcypher.com` are such sites.

We stress the fact that we throw to the blockchain not the data itself, but an encrypted hashed, so that we store a proof of data. Emails, consent status, protocol versions and all related informations won’t appear in clear, these informations are encrypted. And so when a validator wants to check an alleged consent status or a specific version of the protocol, than he has to use the public key and verifies the concordance between the claimed data and the transaction stored in the blockchain as explained in the previous paragraph.

Besides, there is an other concern about the user authentication, but we will go this point in the next answer.

Besides, from a more in-depth technical point of view, transactions stored inside bitcoin blocks are stored following a powerful data-structure called a Merckle Tree, this corresponds to a tree with, at the bottom, transactions that are hashed by joint pairs as and when the hashing scheme bubbles up to the top of the tree where one finds the so-called Merckle root. Hence, this enables verifying that alleged data lies indeed in some transactions by adding the hash value of the Merckle root in a consistent and efficient way. As for the ChainScript, we grouped together all the transactions in order to validate them as a whole, which enables to be cost-savy and not expense bitcoin each time a user triggers an action on our web-platform.

We updated the manuscript in order to make more clear what we mean by publically verifiable.

[1] https://bitinfocharts.com/

**This then boils down to the physical identity crisis, which in itself can be a separate research manuscript. The authors can add a paragraph explaining the limitations of this crucial drawback in detail. So far there is only a brief description in the manuscript. Additionally, a public ledger may be unpopular with patients for enrollment. A detailed explanation on what ‘public’ means and which entities will be able to see the participant’s consent data from the distributed ledger needs to be highlighted.**

In the context of this POC, we are aware that our solution is functional but do not tackle the question of ensuring user detains keys authentication engaging directly the blockchain transaction when submitting action on the website, since this was not our primary concern. And we agree that this could give us the opportunity to go through a new research manuscript. First, we want to separate 2 issues that are different kind of concerns. 

- The first one is the authentication by itself, i.e. ensuring that the person presently answering is precisely the person that is supposed to do so. If for sure this is an important issue, this is related to the online nature of the consent and not the specifics of our blockchain implementation. This subject is also known as KYC problem, Know Your Customer. Lots of services face this issue, such as banks, public institutions for online administrative process, tax payments for instance and many solutions are currently deployed and we won’t focus on them. 

- The second one is related to the fact that cryptographic key pairs are generated by a third party, which for sure is not fully satisfactory. This can find solutions that we could test in a new implementation. Here’s how 

1) One way to tackle this issue can consist in storing the keys in physical objects, such as USB keys, protected by a password or a PIN code [1]. There are plenty of such material bitcoin wallets that allow storing the key in some material, in so called cold or hot storages. Examples of cold storage are paper wallets that suppose to flash QR code to get back private key,  there are also specific hardwares that enables the off-line decryption/encryption with a private key. Hot storage may enable keeping the pair of keys on a computer without protecting them from the online environment, which is still secure and which we believe to be sufficient in our case. Since in a real life clinical trial, there is a meeting point between subjects and investigators, one of these materials could be given by hand to the subjects. 

We can also choose a process where keys could be generated by the user and stored in the their web browser, and this without the user having to worry of the complexity of this process generation and storage. Indeed, some platform registration “connect-button” can trigger the event of generating the pairs and attach them to the local instance of the user: either directly in the web browser, the principle being the same as some bitcoin wallet provider for instance for Chrome browser, such as `KryptoKit Bitcoin Wallet` or `Bitcoin Cash Wallet`[2]. We mention here the converging efforts of main browser providers such as Chrome, Safari, Firefox to build-in a currency-agnostic web payment standard, which supposes to bring the keys there, in the browser [3]. We can also implement a local file stored in the desktop that the subject would have to use each time he or she wants to triggers a action that will be the subject of a transaction, and this can advantageously complement the browser solution in case the user changes web browser. Depending on the design of the study, the online platform can be accessible by anyone or we can send an email with a link bound to a token to each participant. 

We believe that storing keys in the web-browser is safe enough in our context and it would have the major benefit for the user not to worry about the key manipulation. 

Any of these solutions enables the subject to throw directly the transaction to the blockchain. 

*remark 1* here we address the question of decentralizing the key management but we won’t be able to address user authentication issue. KYC has plenty of solutions that are always pretty heavy, such as sending ID pictures and we can of course implement one of them. However, if we implement some smart contract and if we make an assumption, which is a strong one, that we get some trusted emails from participants then there might be a way to enforce the process though still having weaknesses and not as secure as a full KYC process. Indeed, we can bind the user email to its public key in order to prevent from the situation where some malicious investigator decides to send transaction in the behalf of some subject and there are ways to check that an email was effectively sent from the alleged sender, for instance by sending email content as an input to a smart contract and analysing the email header : the user logs into the platforms, a key pair is generated, moreover a transaction is sent binding some metadata consisting in the public key, the user email and some id corresponding to a study identifier, a smart contract checks the consistency of the email content and binds in the blockchain some public key and an email. That way, we improve the one-to-one binding between one public key and a subject email and this tracks user transactions corresponding to the ongoing study. We mention also that there are other popular blockchains, such as Namecoin, that enables the binding between keys and some other data, such as DNS, emails or other metadata. But here, we want to enforce that the study subject effectively sends the transactions. 

*remark 2:* let’s note that regarding the transaction binding key, email and study id, we can proceed on a dedicated blockchain, called Namecoin, which is a fork of the bitcoin blockchain and especially devoted to link names and keys, in fact and historically DNS and keys. Here, we see again that adding a study identifier is important since user may already have a some keys attached to their email.

*remark 3:* we want to stress the fact that even some malicious investigator consented in behalf of a subject, this very subject would still have to show up to participate to the study if this is not an online study. 

*remark 4: *on principle, patient invention falls beyond the scope of the Blockchain. However, we want to mention that, in the future, if we get a way to bind biometric attributes to public keys detainers, than we higher the barrier to patient invention. For instance, supposing we can generate some pair of keys through some `TouchID` Apple-like button, hence through fingerprints that are converted in pair of keys - of course this would happens on the user side -, then in case of patients invention, the fraudster would have to prove that the keys are derived through fingerprints, which suppose to recruit as many accomplices. We mention here `tokenise` solution [4] that is currently under conception and which can be interesting in this spirit.   

2) The second step is to fund the bitcoin account as soon as they are created so that each user can validate their transactions with their pair of keys. Of course, the public keys will be known at the account creation and let then the stakeholders send the correct amount of bitcoins in order for the user to process his transactions.

Though technically speaking this implementation is fully doable, one of its drawback is that the transactions are no longer grouped and one of the advantage of our current solution is that it enables the grouping of user interactions and validate them in a whole block of transactions. This is where Smart Contracts come into play. Of course, Bitcoin offers the possibility to build smart contracts, but they are quite constrained, indeed BitScript is stack-based language devoted to do some very specific tasks and is not very flexible to conceive custom smart contracts. 

This being so, we could turn to the solution of Ethereum, which is a popular blockchain and which offers a full flexibility in the use of Smart Contracts. Moreover, the high level framework allows a friendly way of coding them. In this setting, the steps detailed above would be the same and the key pair generated could be for instance stored in the web browser, but then the transactions grouping could be entirely ensured through a Smart Contract. Indeed, each user event could be queued and then depending on some settable parameters, the transaction on the Ethereum blockchain would happen globally for all the transactions when enough of them are queued. However, this Smart Contract implementation is not that simple and we’ll turn back to that question later;

At last, we believe that current state of the implementation is directly usable for clinical trials the way they are conducted today, and this could be a first step improvement. Sure, users won’t control their keys the way we would want and this suppose that some stakeholders have root-permissions to create key pairs to be trusted. But, the current process of clinical trials heavily relies on the existence of such stakeholders such as investigators, sponsors, regulatory agencies or IRBs, and if we consider the most trustable of them, we can still benefit from the robust, verifiable characteristics of blockchain technologies but with a supplementary point of trust towards a third party. For instance, to prevent from key forgery, external party under the supervision of the sponsor, such a regulatory agency or the IRB could play the role of forwarding the user interaction through transactions, and since user would access their pairs of keys, we could plan to give them the opportunity, if they find it necessary, to verify in an ergonomic fashion the status of their transactions in the public blockchain.  

We updated the text accordingly in order to precise how we can tackle the issue of key management.

[1] https://bitcoin.stackexchange.com/questions/19646/wallet-advice-for-users-without-technical-knowledge

[2] https://chrome.google.com/webstore/search/Bitcoin%20Wallet

[3] https://www.w3.org/blog/wpwg/2017/09/14/payment-request-api-now-being-implemented-in-all-major-browsers-advances-on-the-recommendation-track/

[4] https://tokenize.com/

**In the introduction, the authors have given examples where the consents have been mishandled. Some of the examples are not specific to the consent mishandling, for example, the DECREASE studies where the concern was of falsifying the data as a whole. The manuscript would look more precise if examples of clinical trials or research studies specific to consent mishandling are given.** 

We did not find a study about which the problems was strictly limited to consent issues, it appears that study stained by misconducts carries a wide range of flaws, amongst which ones related to consent. We added some reference [1] about frauds in clinical trials and where the author reports the issue of backdating consent document and indicates that the most commonly fabricated documents are patient diaries and informed consent Forms. In [2] reports mishandlings of consent documents that were backdated. We refer also to the FDA report that is referred in [2] but no longer accessible on their website but we found here [3] 

Our blockchain implementation in order to improve the consent process finds its rationale in studies reporting issues compromising the consent process quality. Indeed, study compiling FDA clinical trials inspections from 1977 to 2009, revealed that 2/3rd of clinicals trials were assessed as carrying misconducts, around 15% of the latter were related to serious consent process flaws, main of which were: failure to obtain written informed consent, failure to provide copies of the document to subjects, consent document not signed or not dated, missing pages in consent document provided to subjects, failure to re-consent when new information becomes available, use of wrong forms, use of expired, non-validated or unapproved forms, the only document last page kept in study files, (reference [3,4] in the manuscript). 

According to [4], we note a decreasing global trend of the issues observed in the setting of FDA investigations: for the period from 2000 to 2009 versus 1990 to 1999, the issues related to consent process in the audited clinical trials have been divided by a factor 4. However, Seife and al. (reference 5 in the manuscript) that emits serious doubts upon the results of FDA [5] and studied clinical trials from 1998 to 2013, and grouping together failure to protect the safety of patient and consents, found that these correspond to more than a half of the issues misconduct in the scrutinized clinical trials.

We updated the text accordingly and added right references.

[1] Gupta, A. (2013). Fraud and misconduct in clinical research: A concern. Perspectives in Clinical Research, 4(2), p.144.

[2] Hazra, A. (2011). Use, abuse and misuse of notes to file. Perspectives in Clinical Research, 2(1), p.38.

[3] http://klinikfarmakoloji.com/files/ketek%20FDA%20mektubu.pdf

[4] Morgan-Linnell, S., Stewart, D. and Kurzrock, R. (2014). U.S. Food and Drug Administration Inspections of Clinical Investigators: Overview of Results from 1977 to 2009. Clinical Cancer Research, 20(13), pp.3364-3370.

[5] http://www.slate.com/articles/health_and_science/science/2015/02/fda_inspections_fraud_fabrication_and_scientific_misconduct_are_hidden_from.html

**Secondly, the authors highlight the empowering the patients and improved enrollment in a clinical trial. It would help if authors expand on patient empowering. From the manuscript’s current structure, the patient empowerment remains at same status as it would be in a written consent, to the extent that patients can pull out of the study whenever they want and can see which entities are involved in the study. Hence, a discussion about this crucial aspect is worth including in the manuscript.**

Indeed, you are right to say that the current implementation of our consent workflow do not directly improve the enrollment rate. But, our point is to focus on trust, and we mean that trust is central to clinical trials engagement. As explored in [1], lack of trust of industry-sponsored clinical trials, and as indicated in this very paper especially the requirements that surrounds consent process, may create confusion or even reluctance to join clinical trials. Moreover, as detailed in [2], given the trend to design clinical studies that can leverage the opportunity of data massiveness and real time, building robust reliable online consent is of substantial importance and can help engaging more people in participating to clinical research from every where they are on the internet. From an other perspective but leading to the same idea, a recent paper published in Nature [3] asserts that privacy and consent are one of the 4 ethical priorities of Neurotechnologies and AI, we think that it is more generally the case for the whole field of clinical research which is going to be more and more tech-intensive. In this very paper, Blockchain and Smart Contracts are announced to be interesting tool to tackle this challenge. We think these tools in bringing more reliability and trust into clinical trials meet patients concerns and can form a solid ground to engage subjects. 

Indeed, Blockchain helps build reliable processes since the decentralized nature of the protocol and the validation of each transaction by consensus is key to achieve this trust. In our setting, each of the user interactions, consent status, protocol versionning, re-consent status accordingly to these very protocol versions, is “proof-checkable” by anybody. 

Culturally and historically, Blockchain technologies were invented to address the very issue of trust towards industries and even public institutions, distributed network were thought as a kind of response to the lack of trust by conceiving an interaction network architecture which do not relies on a single trusted third party, as credible as it might be, but on distributing to each node of the network a granular part of trust, so that trust compromising would suppose an unfair agreement involving strictly more than a half of the network participants, which occurrence lowers fast when the number of nodes increases. We believe that at least on principle, Blockchain technologies in distributing securely proof of data and by providing technological guarantees to consolidate a network of trust, unite the conditions to find a tool to restore confidence in building consent process reliable and getting patients to join. 

We updated the text accordingly in order to stress the fact trust is the critical pivot to engage subjects in clinical trials, and Blockchain is precisely a technology that was designed to enforce trust. We re-affirmed this point at the beginning of the Discussion section.

[1] Transforming Clinical Research in the United States: Challenges and Opportunities: Workshop Summary. Institute of Medicine (US) Forum on Drug Discovery, Development, and Translation.

Washington (DC): National Academies Press (US); 2010.

[2] New England Journal of Medicine. (2017). Informed Consent — NEJM. [online] Available at: http://www.nejm.org/doi/full/10.1056/NEJMra1603773?query=featured_clinical-trials

[3] Yuste, R & All (2017). Four ethical priorities for neurotechnologies and AI. Nature, 551(7679), pp.159-163.

**Thirdly, the general descriptions of blockchain (ie, 'a giant public datastore') are not entirely correct, and the strengths of the technology ('backbone of the circulation of digital assets, powering any kind of services…') are overstated.**

As, for the statement ` *backbone of the circulation of digital assets*`, if we appreciate blockchain at a generic level, one can draw our attention to Smart Contracts, and these enable the conception of wide range of generic services that supposes contract between parties agreeing on “computer-encodable” terms.

On Bitcoin blockchain, transactions are executed through some scripts, coded with a stack based langage called bitscript. The scripts are kind of rudimentary Smart Contracts, and they open the possibility to have interactions beyond the bitcoin exchanges : smart property, lotteries, proof-of-knowledge. More interestingly, an other popular blockchain, Ethereum relies heavily on Smart Contracts, these are contracts algorithmically implemented on the top of Blockchain and fix rules about which parties agree on, and in our context fixing as specific as needed criteria. A very wide range of services are now provided through Smart Contracts : vote, insurances contracts, data sharing, land property, domain name system for the internet, electronic health records even marriage contracts. 

We meant by blockchain becoming some backbone of digital assets the idea to enlarge the purpose of Blockchain beyond cryptocurrencies and manifest that the applications can be quite diverse. However, we understand that this expression is too general and we updated the manuscript accordingly.

**Furthermore, the unfalsifiable nature of any particular blockchain depends on a distributed deployment, as in many implementations, the person/group owning a majority of the miners could change or alter data. Authors should elaborate on these specifics so that the readers have the understanding of the specific nuances of blockchain. Finally, many of the concerns and issues with consent noted in the introduction (not obtaining IRB approval of new consent forms, etc) are administrative issues – auditing of these updates could be done with the system presented here, but use of blockchain alone would not necessarily prevent all of these issues from occurring.**

As for the resilience of the blockchain to adverse attacks, the most natural one we can think of would be to acquire some more nodes in the network and then win the game since the attacker would have a computing power advantage as for the Proof-of-Work validation, this is called the sybil attack. But, the larger the network grows, the harder is the difficulty of the Proof-of-Work problem solving, so these kind of attacks are easily defeated. In the spirit, a most well-known attack is the so-called 51% attack, when some adversary gets more than a half of the computing power. This is not to be excluded but never happened successfully so far on the Bitcoin Network. Here, we want to precise that even if such a situation happened, then first there won’t be any chance for an attacker to steal bitcoin, because each transaction supposes the digital signature of the sender, which in turn supposes to be in a possession of the related private key, second this would prevent such an attacker to continue gaining money through mining, since the “double spend” attempts would be noticeable, and so the bitcoin value will fall, and  there won’t be any interest to continue.

Besides, Blockchain is not a one-size fits-all solution and some issue can’t be addressed by the blockchain alone, but as to the issues stated, using blockchain enables to keep track of the consent document, to version it through timestamping, to control its format, its consistency and its immutability, to ensure subjects’ consent and re-consent status with respect to changes of the protocol, and to make all the process “proof checkable”, so that failure to obtain written informed consent, failure to provide copies of the document to subjects, consent document not signed or not dated, missing pages in consent document provided to subjects, failure to re-consent when new information becomes available, use of wrong forms, use of expired, non-validated or unapproved forms, the only document last page kept in study files, falls in the scope of what can be better controlled by Blockchain technologies. Binding hashed of the protocol and its versions to the consent, preventing from backdating and giving not only a timestamping but a trusted one strengths the consent process.

We updated accordingly the manuscript. 

**In the methods section, the authors argue on why they opted for bitcoin blockchain (because of stability, immutability, large mining network). Given that the authors rely on smart contracts for future developments, it is difficult to understand why they did not opt for the Ethereum which has the same benefits as those of bitcoin blockchain with added benefit of smart contract platform. The manuscript would improve if the authors give more context on their choice, and also, what they envision would be used in a real-world application of their POC in a clinical trial.**

The choice of bitcoin was motivated because it is a robust network, born in 2009, and because it allowed us in a rather simple manner to group the transactions in order to save the eventual clinical trials of the cost of the validation of each transaction. Moreover, though we did not use that feature, Bitcoin allows transaction taking multiple inputs (and in fact multiple outputs), and so requiring multi-sig signatures. We think that it could be an interesting feature when put in a context of a real life clinical trial where user would store on their side the pair of keys and would help group the transactions and validate them as a whole. 

Ethereum is more recent, it was released in 2015 and indeed we are definitely sure that it can host transactions we want to achieve. But in order to group the transactions, it would have required the implementation of more complicated Smart Contracts, although Smart Contract has a major benefit in that it enables a great flexibility when mastered. Indeed, Ethereum do not allow multiple inputs and to achieve our goal, it would have needed two contracts, the first would have register some transactions, when some amount of ETH (the ethereum money) or some number of transactions would have reached a pre-defined threshold, and then send the ETH to a second contract.   

We have updated the manuscript in order to motivate more clearly our choice. Besides, we refer the reviewer to the detailed first answer of this document as to a possible real life implementation.  

**The authors state in prior reviews that the exact chaincode/blockchain data cannot be shared for review purposes due to the presence of private information. While this is undoubtedly true, it is difficult to understand, based on the current description, how the authors envision deploying a decentralized system if this is an issue.**

In the current implementation, the current bitcoin transactions can absolutely be made public since any entries are hashed and so encrypted. However, the ChainScript, which is the master document holds the raw data of the consent process. If some stakeholder wants to claim that these are the true data, then any regulator or any body in charge of the verification of the sincerity of the data, either a regulatory, a stakeholder, a publisher willing to verify them, is in a situation to prove the data sincerity on public website, as `blockchain.info` or `live.blockcypher.com` (cf. the above response to the question you raised about the “ *‘publically verifiable’ *” nature of the data, or to be more precise the proof-of-data).

**We agree with concerns from other reviewers regarding how much of a proof of concept was implemented, and concerns with key steps of the process such as identify verification. While it is understood that this is a proof of concept, it would be helpful as a method paper to at least detail how these issues would be resolved in an actual application deployed beyond the proof of concept stage.**

We detailed in the answer of your question beginning with “ *This then boils down to the physical identity crisis...*” how we would deal in a real life clinical trial. It appears that there are different interesting reasonable solutions : to summarize, one way is to equip them with USB keys, already available on the market, that could be sent or given by hand by investigators when meeting the subjects, this USB key is then plugged in any user device and let them activate their transaction with a simple 4 digits PIN code, an other by directly attaching the pair of keys to his browser. Then these bitcoin addresses would be funded by the stakeholder in order for the user to proceed the transactions. 

A second concern is related to the choice of the blockchain network. Bitcoin seems to us a good and robust choice, however if we want to go to further implementation, such as automating the re-consent process when protocol is the subject of major changes, then Ethereum can be a relevant choice in the sense that it allows benefit of the full flexibility of Smart Contracts. Besides and for the record, we mention a possible alternative with regard to the blockchain network: we could think of using permissionned blockchain, or even private blockchains that we briefly evoked in the manuscript, but we believe that public blockchains unite conditions of trust, since nodes participating to the validation are not selected by any third-party.

We updated accordingly the manuscript by indicating the range of solutions that we envision as for the public/private key management and the choice of the Blockchain network.  

**Another aspect of the blockchain important to discuss is the cost effectiveness. Blockchain is resource intensive technology which requires a lot of computational power. Given that the funding of the many research studies is limited, a calculated investment is always required. If authors can talk a little about the logistics and cost effectiveness of this blockchain setup, it would bring a new dimension to the manuscript.****

As mentioned sooner in these responses, we took a great care to integrate by design the cost-effectiveness. This is the reason why we build a data-structure allowing to group transactions in order to limit the cost. We can host in one binary tree called a Merkle tree hundred of thousands of hashes represented by their Merkle root and these informations are stored in a bitcoin transaction. For memory, the transaction fees are around 0.0015 BTC to 0.0016 BTC, depending on the priority of the transactions and we can group transactions by slices of 1000 or 10.000 batches of data, or even more, so that even for large clinical trials the cost would be controlled to the level of some milli-bitcoins. We refer the reader to [1] to check the cost of the bitcoin fees

From an other point of view, there are currently many implementations that tries to tackle the mobilization of such energy consumption in a context of global environment issues. There are Altcoins, that uses Proof-of-Storage instead of Proof-Of-Work, so that the transaction fees consists in accepting to fill some disk space rather than consuming CPUs processing. Some of these, as StoreJ or file.io are quite popular and one can consider them as interesting blockchain on the top of which one can build advanced processes. Ethereum announced their next implementation, called CASPER, based on a Proof-of-Stake rather than Proof-of-Work consensus algorithms, which propose to replace the provision of CPU power by a economic contribution depending on the size of a deposit.

We updated the manuscript to tackle this issue. 

[1] https://live.blockcypher.com/btc/

**Finally, the manuscript can improve on the language. Even after three revisions, there are typing and grammatical errors which are concerning. Manuscript can improve a lot if the authors can thoroughly proof read the manuscript and revise accordingly.**

Thank you for drawing our attention to this point, we updated our manuscript and corrected typing and grammatical errors.

# Peer review: Referee response for version 3

**DOI:** 10.5256/f1000research.12989.r26850

**Published:** 2017-11-01

**Authors:** Jonathan C. Craig

**Affiliations:** 1 Sydney School of Public Health, University of Sydney, Sydney, Australia

This article addresses a critical element in the research process that involves humans. Their goal of adapting a technology for use in clinical research for the purpose of consent is laudable and to be encouraged. Innovation is desperately needed. The authors may wish to consider the following as they iteratively seek to improve their paper

 1. The centrality of consent is incontrovertible. What the authors propose here is a radical solution that has enormous infrastructural and resource implications. The paper would be more compelling if the authors demonstrated that there was a systematic problem in how consents are currently obtained and that, in theory at least, that this method would address these problems, in particular the issue of the need for changing consents.

 2. The IT requirements around clinical trials in particular is already substantial. The notion of a parallel process for all of the data management systems and then for consent sounds particularly daunting. Is it possible that the two systems could be integrated? Otherwise I fear that this approach has limited feasibility.

 3. I'm sure the authors would agree that a trial among a group of research-literate participants is very different to typical research participants. One critical element around proof of concept is not the IT/software capability but whether participants would even use such an approach once, let alone multiple times. As it stands the current study is pre-proof of concept perhaps.

I have read this submission. I believe that I have an appropriate level of expertise to confirm that it is of an acceptable scientific standard, however I have significant reservations, as outlined above.

# Response

**Published:** 2017-11-15

**Authors:** mehdi benchoufi

**Affiliations:** Hotel-Dieu Hospital, Paris Descartes University, France

Dear reviewer,

Thank you for these precious remarks and letting us have the possibility to improve the quality of our work. Hereafter, we provided responses to the raised issues

*1. The centrality of consent is incontrovertible. What the authors propose here is a radical solution that has enormous infrastructural and resource implications. The paper would be more compelling if the authors demonstrated that there was a systematic problem in how consents are currently obtained and that, in theory at least, that this method would address these problems, in particular the issue of the need for changing consents.*

The rationale to implement blockchain solutions to improve the consent process is based on issues undermining the quality of the latter, these were for instance reported by FDA. Indeed, a study compiling FDA inspected clinical trials from 1977 to 2009, revealed that 2/3rd of clinicals trials were misconduct, around 15% of the latter were related to serious consent process flaws, main of which were failure to obtain written informed consent, failure to provide copies of the document to subjects, consent document not signed or not dated, missing pages in consent document provided to subjects, failure to re-consent when new information becomes available, use of wrong forms, use of expired, non-validated or unapproved forms, the only document last page kept in study files, (reference [3,4] in the manuscript). However, we note that the global trend is the decrease of the issues observed in the setting of FDA investigations, for the period from 2000 to 2009 versus 1990 to 1999, the issues related to consent process in the audited clinical trials have been divided by a factor 4 [1]. From a similar perspective, studying clinical trials from 1998 to 2013, Seife and al. (reference 5 in the manuscript), grouping together  failure to protect the safety of patient and consents, found that these correspond to more than a half of the issues misconduct in the scrutinized clinical trials.

In this context, using blockchain enables to keep track of the consent document, to version it through timestamping, to control its format, its consistency and its immutability, to ensure subjects’ consent and re-consent status with respect to changes of the protocol, and to make all the process “proof checkable”. Indeed, one core functionality is unfalsifiable timestamping, so that our implementation outputs a single master document accounting for the whole consent process and if any single datum would be corrupted then the whole consent would be broken. This master document can then be checked in any public website offering a service consisting in verifying the correspondence between data and its hashed counterpart, i.e. its proof of existence stored in the blockchain.

We would also like to underline some additional points which justify in our view the potential of blockchain and smart contracts for consent, as we have implemented her. Indeed, in a more prospective way,

- we anticipate that Smart Contracts which are contracts algorithmically implemented on the top of Blockchain and fix rules about which parties agree on, and in our context fixing as specific as needed criteria that would account for what clinical trial stakeholders consider as consent process quality rules, could help automate a large part of consent process validity checking. For instance, we can realistically bind *automatically* any change in the protocol to the patient re-consent seeking, the latter being a vast subject of concern (reference 1 in the manuscript). 

- besides, there is strong trend of research to build clinical studies design that can be adapted to the massiveness and real time of data. In this context as developed in [2], building robust reliable online consent is of substantial importance and can help engaging more people in participating to clinical research from every where they are on the internet. In the view of developing observational studies based on the analysis of real life data, developing reliable online consent is of importance. Indeed, we live in a current era of massively produced data, by 2020 one will count 20 billions devices and according to some studies, by 2030, 500 billion devices and objects will be connected to the internet [3,4]. In addition to the 2.5 exabytes of data produced each day, i.e. 10^18 bytes of data, the expected billions of connected objects will receive and produce data in the so called IoT, and this can be considered as many data that can have a potentially health significance given the power of new algorithms and artificial intelligence (AI) techniques derived from Machine learning. There are current implementations of Blockchain specifically designed for the IoT network, such as IOTA [5] and there is a lot to be done to build consent processes adapted to these kind of studies.

A recent paper published in Nature [6] asserts that privacy and consent are one of the 4 ethical priorities of Neurotechnologies and AI, we think that it is more generally the case for the whole field of clinical research which is going to be more and more tech-intensive. In this very paper, Blockchain and Smart Contracts are announced to be interesting tool to tackle this challenge.

- From a general point of view, as explored in [7], lack of trust of industry-sponsored clinical trials, especially the requirements that surrounds consent process, may create confusion or even reluctance to join clinical trial.

But, culturally and historically, Blockchain technologies were invented to address the very issue of trust towards industries and even public institutions, distributed network were thought as a kind of response to the lack of trust by conceiving an interaction network architecture which do not relies on a single trusted third party, as credible as it might be, but on distributing to each node of the network a granular part of trust, so that trust compromising would suppose an unfair agreement involving strictly more than a half of the network participants, which occurrence lowers fast when the number of nodes increases. We believe that at least on principle, Blockchain technologies in distributing securely proof of data and by providing technological guarantees to consolidate a network of trust, unite the conditions to find a tool to restore confidence in building consent process reliable and getting patients to join.

We updated the manuscript in order to add some consent process issues, for example "failure to provide copies of the document to subjects", and precise that while issues regarding the consent process in clinical trials are substantial, we notice in the same time that the global trend of FDA studies shows a decreasing trend of the so called OIA, and this concerns consent as well.

Besides, in a context of an extreme sensitivity about right to informations, privacy respect, building a consent process as irreproachable as what could be achieved using the current state of knowledge and technologies, is critical: trust is the critical pivot to engage subjects in clinical trials, and Blockchain is precisely a technology that was designed to enforce trust. We re-affirmed this point at the beginning of the Discussion section. Besides, again in Discussion section, we updated the document in order to put in perspective that quality of consent process is critical, especially in a remote setting given the place that should take online studies in current Big Data and connected devices era.

[1] Morgan-Linnell, S., Stewart, D. and Kurzrock, R. (2014). U.S. Food and Drug Administration Inspections of Clinical Investigators: Overview of Results from 1977 to 2009. Clinical Cancer Research, 20(13), pp.3364-3370.

[2] New England Journal of Medicine. (2017). Informed Consent — NEJM. [online] Available at: http://www.nejm.org/doi/full/10.1056/NEJMra1603773?query=featured_clinical-trials

[3] Internet of Things report, Cisco https://www.cisco.com/c/r/en/us/internet-of-everything-ioe/internet-of-things-iot/index.html

[4] https://www.gartner.com/newsroom/id/3598917

[5] IOTA, https://iota.org/

[6] Yuste, R & All (2017). Four ethical priorities for neurotechnologies and AI. Nature, 551(7679), pp.159-163.

[7] Transforming Clinical Research in the United States: Challenges and Opportunities: Workshop Summary. Institute of Medicine (US) Forum on Drug Discovery, Development, and Translation.

Washington (DC): National Academies Press (US); 2010.

*2. The IT requirements around clinical trials in particular is already substantial. The notion of a parallel process for all of the data management systems and then for consent sounds particularly daunting. Is it possible that the two systems could be integrated? Otherwise I fear that this approach has limited feasibility.*

We think that the consent process we suggest can be without hassle fully integrated in a data management system. Indeed, it can be wrapped up as a plugin or even more simply accessible from an API - Application Program Interface. Indeed, the main issue is to access a notarization tool from any data management system. In our implementation, in the `README.md` file -https://github.com/benchoufi/DocChain/blob/master/README.md- we refered to an API handling the bitcoin interactions, exposed through URL such as https://docchain.stratumn.net/some/path/to/some/services. This gives an endpoint to connect a data system to blockchain transactions. 

Besides, if one wants to build such a tool from scratch, it needs expertise but can be done : in substance the core part of the software logic relies on the notarization process, which deals with blockchain transactions, precisely the possibility to put on a blockchain the proof of informations which are believed to be critical and about which we want to ensure sincerity. Since all the pieces of softwares to build such a solution are largely available in Open Source, the required IT tools can be found easily. However, to build a solid, professional notarization tool, keeping track of protocol versions, and compliant with a careful use of the bitcoin valued transactions that can be costly if considered in a context of a large clinical trial, then this requires more advanced skills. For instance, we make use of specific data-structures which enables to group together transactions and then validate them in a same pool. This has the advantage of using with parsimony the blockchain bitcoin-costly transactions though preserving the integrity of the data, since any bit of data in this data-structure would have been flawed, the entire data-structure would be broken. Again, a tool that would be build up in this manner could be fully integrated to a current data management system, blockchain interactions would be designed as API calls to services.

For the record, we indicate in the manuscript that the transaction fees vary between 0.0015 BTC to 0.0016 BTC, depending on the priority attached to these transactions. In our setting, thanks to the data-structure, wich is a binary tree called Merkle tree, we can manage hundred of thousand of hashes in a single transaction. Then, in a live clinical trial; we can group transactions by slices of 1000 or 10.000 batches of data, or even more, so that cost can be controlled to the level of some milli-bitcoins. We refer the reader to [1] to check the cost of the bitcoin fees

With regards to the mentioned expertise for developers to build such tool. We want here to stress two facts

- in the upcoming months, blockchain programming tools will to be considerably simplified thanks to the current great effort led by open source communities to abstract out all the complexity into frameworks. We can draw a consistent parallelism with the web tools which were rather complicated in the mid 90's and that were rapidly proposed through high level frameworks usable by anyone including anybody not familiar with web development but able to click-and-deploy webiste, being only concerned by adding some text and image contents.

- a good example to build a notarization service that would not require so much expertise would be to access the notarization process from services that offer ready-to-use APIs. A good example of such a service can be found in [2].

[1] https://live.blockcypher.com/btc/

[2] https://poex.io/developers

*We updated the document and proposed a `Guide to installation` document in which whe detail the tools needed to reproduce the experiment.*

*3. I'm sure the authors would agree that a trial among a group of research-literate participants is very different to typical research participants. One critical element around proof of concept is not the IT/software capability but whether participants would even use such an approach once, let alone multiple times. As it stands the current study is pre-proof of concept perhaps.*

Thank you for giving the opportunity to say that what we consider to be interesting in the current setting is that the solution is almost ready to use in a production setting. Indeed, the blockchain complexity is totally hidden from the interacting user. So that in practice, from a user point of view, there is no burden in the front-end website interactions, though they should be informed that technical - blockchain - guarantees have been taken to ensure transparency and security of the consent process. None of the complex technical aspects interferes with their experience. The process is transparent for the user in the sense that all the complexity of blockchain transactions is taken care by the back-end server. However, the essential part of the issues that can be raised are related to the online nature of the implemented process.

As mentioned in your comments, what we considered as a proof-of-concept needs to be customized and validated in real context. Our work needs a feasibility study that we will to conduct. And, two main points may be worked out in a real production clinical trial

1. Keys authentication forwarding issue

In the context of this POC, we are aware that we implemented a functional solution about which we did not tackle the question of ensuring user detains keys authentication engaging directly the blockchain transaction when submitting action on the website, since this was not our primary concern. In a production setting, we could go through storing the authentication keys on line, or to store them in physical objects, such as USB keys, protected by a password or a PIN code. There are plenty of  so called Hardware Wallet [1].

In this spirit, we believe the simplest way to improve user authentication is to store keys in the web-browser. We think that it is safe enough in our context and it would have the major benefit for the user not to worry a single time about this authentication process. Besides, this would suppose that the stakeholders fund the bitcoin account - or anly Blockhain related network account - of each subjects in order for them to process directly the transactions. Moreover, using Smart Contracts network, for instance on ethereum, these very transactions could be grouped together in a simple manner.

However, we believe that the current state of the implementation is directly usable for clinical trials the way they are conducted today. Indeed, we won’t deal in that case with advanced double keys user authentication process. This supposes that some stakeholders have root-permissions to create key pairs to be trusted. The current process of clinical trials heavily relies on the existence of such stakeholders such as investigators, sponsors, regulatory agencies or IRBs, and if we consider the most trustable of them, we can still benefit from the robust, verifiable characteristics of blockchain technologies but with a supplementary point of trust towards a third party. For instance, to prevent from key forgery, these could be generated by the sponsor or any other external party under the supervision of the sponsor, such as regulatory agency or the IRB, and given by hand to the patient in an USB keys or any other support along with information documents through.

2. ensure that the informations have correctly been understood by the reader. Though this is not related with the blockchain based tool but the on-line nature of the online consent tool, we believe that there could be improvement by ensuring the correct understanding of the user by first proposing a pedagogical presentation video and suggesting him a quizz after the reading of the protocol he is about to consent or not. There are bunch of tools and references on the subject. Here's some :

*We updated accordingly the manuscript to signify that no peculiar expertise is required to make use of the tool. However, we indicate that the current process can be strengthened, especially to ensure subject understanding of protocol leading to an improved consent decision.*

[1] https://bitcoin.stackexchange.com/questions/19646/wallet-advice-for-users-without-technical-knowledge

# Peer review: Referee response for version 3

**DOI:** 10.5256/f1000research.12989.r24031

**Published:** 2017-07-31

**Authors:** Daniel S. Himmelstein

**Affiliations:** 1 Systems Pharmacology and Translational Therapeutics, Perelman School of Medicine, University of Pennsylvania, Philadelphia, PA, USA

After the previous two rounds of revision, there are still major outstanding issues. The revisions only minimally react to my previous reviews and do not include the substantial changes that would be necessary for me to approve this study. For context, see the changes from version 2 to 3 here.

 Here are the main factors weighing on my decision:

1. The digital signature scheme that was implemented is worthless in terms of proving that a participant consented. Hence, I entirely ignore and discount this aspect of the proposal.
2. The "proof of concept" justification provided by the authors for the methodological shortcomings and incomplete nature of their study is frustrating. The authors make statements such as (manuscript typos bolded):
  > The current implementation is an application of the **frst** principle. Ideally, we would have to build a patient authentication system which **does not relies** on any trial **stackholder**
  > It would be possible to build a Smart Contract that will be executed with the only condition that patients will only be included when the **enrolment** is complete.
  The assertion that solutions to these problems will come later is insufficient. The authors have to implement the solutions or cease promoting their benefits. In my opinion, these are unsolved problems. Implementing such solutions is difficult: suggesting that a solution exists but not offering an implementation is therefore worthless.
3. The study continues to overstate its reliance on blockchain technology. As our previous dialogue has confirmed, the study only uses the a blockchain for timestamping. Timestamping is an important addition, but does not justify titling the study "blockchain protocols in clinical trials" nor the abundant discussion of blockchains when their role is relatively minor in the actual proposal.

To help explain my decision, I'll elaborate on the theoretically sound aspects of the authors' proposal. The following workflow is theoretically sound, albeit poorly communicated in the manuscript:

1. A webapp is created to administer an electronic consenting process. The webapp can be designed to enforce a rigid workflow that ensures the right steps are performed in the right order.
2. The user interactions and inputs from the electronic consent process can be recorded via a Chainscript JSON file. Therefore, one can store the digital equivalent of paper forms from a traditional physical consent process.
3. The Chainscript JSON file can be timestamped using the Bitcoin blockchain. This prevents predating the existence of a consent record.

Now while a clear, compelling, and clean implementation of the previous steps would be of interest, the study fails to achieve this. Specifically, the webapp is not publicly hosted, so users can experiment with and observe the proposed electronic consent implementation.

 Second, the authors do not provide any Chainscript JSON files for their study. In their previous response to my review, they link to a Chainscript file unrelated to their study. Even more troubling is that their Chainscript example from the manuscript appears to be manually edited from an example Chainscript document rather than computer-generated output from their consent application. As I mentioned in previous review, the JSON example contains flagrant formatting errors suggesting it was created by hand. Furthermore, the Chainscript includes a mapID of 56c11d0ea55773bc6e681fde. The same mapID is also used in the Stratumn documentation, suggesting copying and pasting from the docs.

 The question remains **did the study actually produce any real Chainscript JSON files or timestamp even a single Chainscript file**. It's telling that the authors still haven't revealed a Chainscript file whose past existence has been timestamped. Hence, there is no evidence that the authors actually implemented their proposed "proof of concept".

 Given the severity of the outstanding issues despite the number of previous rounds of review, I do not intend to review this study again. Hence, my decision to not approve this study should be considered final.

I have read this submission. I believe that I have an appropriate level of expertise to state that I do not consider it to be of an acceptable scientific standard, for reasons outlined above.

# Response

**Published:** 2017-08-16

**Authors:** mehdi benchoufi

**Affiliations:** Hotel-Dieu Hospital, Paris Descartes University, France

Dear reviewer,

We are surprised by the serious charges raised against the proof-of-concept work we present. There appears to be a misunderstanding. Actually, there seems to be doubts questioning whether “the study actually" did "produce any real Chainscript JSON files or timestamp even a single Chainscript file”. **This is not true: we produced a JSON file which is provided in the text.**

1) If you look carefully at the material we furnished, the figures 4,5,6 corresponds to the data structure we got from the production experimentation and these correspond to the validated transactions. Moreover, we let as an example a JSON data structure where we listed different entries, as any documentation oriented information, and we picked up an example file and structured it accordingly to the experiment purpose. And so the HASH ID ` 56c11d0...` is not "flagrant errors" and is set as an example inside this merkel tree data-structure. So, if one wants to check the integrity of the transactions hashIDs, one should consider those appearing in the figures 4, 5, 6.

We have sent to the only editor the production Chainscript file, according to the editor instructions. Of course, the names and emails are blanked out, to respect data privacy as requested by *F1000Research.*

2) We insist with the fact that we identified linking digital identities and physical entities as an important issue and as explained in our series of responses to the reviews :

- We recall that major and serious issues identified by the FDA corresponds, by a proportion of about 10% of trials, to failure to obtain written informed consent, consent documents not signed or dated, missing pages in consent document provided to subjects, failure to re-consent when new information becomes available, use of expired, non-validated or unapproved forms. This is the context we are embedding in our work. 

- **We want to provide solutions coherent with regards to the current state of clinical trial usages** and not an idealistic one. So that, letting the investigators create keys seem not, to our point of view, the subject of major concern. Indeed, the heavy part numerous bias that entail research quality and clinical trials reproducibility are related to a posteriori reconstruction or untraceable missing information. We already furnish examples such as  statistical analysis plan, definition of outcomes, inclusion and exclusion criteria, secondary effects traceability… 

- Besides, this issue has to be raised in “production”. We are here proposing a POC and we already suggested in the preceding round of revision that we could provide for the patients a proper generation key online interface, and this would be doubled by a key registration with a material object :  USB cards are now provided by some vendors.

Despite, most of the questions we are asked are focused on Blockchain technology, **one should have in mind that this is not a technology paper but medical research one.**

  

3) Regarding the workflow that we mentioned and developed in three points, we would like to emphasise that is exactly what we have done : 

- input and users transactions where recorded.  

- the electronic consent process is “blockchained": the consent status is set into the Chainscript. Moreover,  any version of the protocol document is hashed, versioned and bound to the consent status. 

- the Chainscript file is timestamped on the Bitcoin network. Chainscript helps group the transactions and validate them as if it were one. The hashes of this data structure form a coherent and consistent group of hashes, any of them would be corrupted results in  invalidating the whole datastructure . This “SideChain” approach enables us to reduce costs of transactions, which is especially useful when dealing with large clinical trials.

I stay at disposal for any further remark. 

Best regards,

Mehdi

# Peer review: Referee response for version 2

**DOI:** 10.5256/f1000research.12384.r22303

**Published:** 2017-05-22

**Authors:** Daniel S. Himmelstein

**Affiliations:** 1 Systems Pharmacology and Translational Therapeutics, Perelman School of Medicine, University of Pennsylvania, Philadelphia, PA, USA

In version 2, the authors updated the manuscript and responded to my review of version 1. I'd like to thank the authors for these additions, which provide a clearer picture of the trust model and proof of process. To assist in my review, I created a GitHub repository that includes a diff between versions 1 and 2.

**Trust model**

 In their response, the authors clarify who generates the private key for a given participant:

 > A participant cannot simply generate any Bitcoin address and use it to sign, because the private key is created on the server by the agent (but not saved on the server), then is sent to the email address.

 I believe "the agent" refers the agent folder of the benchoufi/DocChain source code. In their proof of concept, the clinical trial investigators host the agent. Presumably, the agent could also be hosted by a trusted third party, such as Stratumn or a governing body like an Institutional Review Board.

 It's crucial to note that the proof of process for participant consent requires complete trust in the agent. Assuming the agent is hosted by the investigators, then we must trust the investigators to have faithfully run an uncompromised agent. Hence, the proposed implementation provides an equivalent trust model to the current consent process. In both cases, one must trust the investigators to truthfully collect consent. Presently, we trust that investigators did not forge a participant's handwritten signature. In the proposed implementation, we trust that the investigators ran an agent that properly generated (and then deleted) a private key for each participant's email. For the time being, the later is perhaps even less verifiable than a handwritten signature. Regardless, the proposed digital identity solution requires a trusted party.

**Proof of process**

 The authors should more clearly document the proof of process underlying their approach. The Chainscript examples (the code block and Figure 4–6) are a good start. However, Figure 4–6 are poor resolution and difficult to read. More discussion of the Chainscript format along with instructions to verify a process and timestamp are needed. For example, there is no example Chainscript JSON file provided with step-by-step instructions on how to verify or evaluate it. The Chainscript code block is littered with broken JSON syntax, so it's certainly an insufficient example. With more details, additional questions may arise. For example, is the 12-byte mapID (with a best-case attack complexity of 2 ^96^) secure against preimage attacks? The manuscript doesn't appear to specify what hash algorithm is used.

**Smart contracts**

 The manuscript is misleading regarding "smart contracts" and implies that the study proposes a smart contract enrollment protocol. Specifically:

1. Figure 1 includes a smart contract step in the workflow.
2. The abstract states "a blockchain core functionality, named Smart Contract, can help prevent clinical trial events not to happen in the right chronological order".
3. The introduction states "This makes it possible to build a Smart Contract that will be executed with the only condition that patients will only be included when the enrolment is complete".

As the authors admit in their response to my review of version 1, they "did not implement" anything related to smart contracts. Implementing a smart contract to manage enrollment is not trivial. Unless the authors actually implement such a contract, they should remove claims about its utility and applicability. Discussing smart contracts in the discussion would be justified, but it's misleading to suggest that the current proposal involves smart contracts.

**Overstated blockchain usage**

 In their response to my review of version 1, the authors clarify that the primary achievement of the study is to create a web-based consent workflow that makes it most natural and easy to perform the proper consent process. However, the study only minimally leverages the guarantees provided by cryptography and secure blockchains. For example, the study does not achieve *trustless consent*, whereby participant consent can be provided and verified in a decentralized manner without having to trust any other parties. However, in the abstract, the authors imply the trustless & decentralized aspects of Bitcoin apply to their consent process:

 > This is a distributed technology that brings a built-in layer of transparency and traceability. Additionally, it removes the need for third parties, and gives participative control to the peer-to-peer users.

 In reality, the only area where a blockchain was applied is for the Chainscript timestamping. I agree this timestamping is valuable for its ability to prevent retroactive consent forgery. However, it's insufficient to verify an actual participant's identify or consent. Foremost, the use of blockchain timestamping is not sufficient to justify the grandiose claims of blockchain relevance to clinical trial consent.

 In other words, the proposed consent protocol would suffer little were all blockchains to immediately disappear. The blockchain is not essential to implement more automated, web-based, and reliable consent processes. Yet the study titles itself "blockchain protocols in clinical trials" and implies that blockchains are what allows "transparency and traceability of consent". The manuscript does not adequately differentiate between speculation and the actual ways in which the study leverages blockchain technology.

 For me to consider approving this study, the authors would need to drastically reduce their claims regarding the benefits of blockchain usage for clinical trial consent applications. In addition, greater clarity and focus on the specifics of their proof of concept implementation would be necessary.

I have read this submission. I believe that I have an appropriate level of expertise to state that I do not consider it to be of an acceptable scientific standard, for reasons outlined above.

# Response

**Published:** 2017-06-21

**Authors:** mehdi benchoufi

**Affiliations:** Hotel-Dieu Hospital, Paris Descartes University, France

**Trust model**

*In their response, the authors clarify who generates the private key for a given participant:*

**

*> A participant cannot simply generate any Bitcoin address and use it to sign, because the private key is created on the server by the agent (but not saved on the server), then is sent to the email address.*

**

*I believe "the agent" refers the agent folder of the benchoufi/DocChain source code. In their proof of concept, the clinical trial investigators host the agent. Presumably, the agent could also be hosted by a trusted third party, such as Stratumn or a governing body like an Institutional Review Board.*

**

*It's crucial to note that the proof of process for participant consent requires complete trust in the agent. Assuming the agent is hosted by the investigators, then we must trust the investigators to have faithfully run an uncompromised agent. Hence, the proposed implementation provides an equivalent trust model to the current consent process. In both cases, one must trust the investigators to truthfully collect consent. Presently, we trust that investigators did not forge a participant's handwritten signature. In the proposed implementation, we trust that the investigators ran an agent that properly generated (and then deleted) a private key for each participant's email. For the time being, the later is perhaps even less verifiable than a handwritten signature. Regardless, the proposed digital identity solution requires a trusted party.*

 Dear reviewer,

 Thank you for these remarks. Indeed, you are right that, in the current setting, the “agent” hosts the entity that generates the set of keys. We have a few remarks regarding this issue.

 The main aim of this implementation is to fight some specific issues related to clinical trials consent process, major concerns documented by the FDA being failure to obtain written informed consent, consent document not signed or not dated, missing pages in consent document provided to subjects, failure to re-consent when new information becomes available, use of expired, non-validated or unapproved forms. So, we simulated several stakeholders, such as investigators, institutional review boards, that played the role of trusted parties who were granted access to a dashboard monitoring all the blockchain transactions. We have considered that patients patients invention and so forging consent is hopefully a rare phenomenon in real life clinical trials, we did not prioritarily focus our attention to the very point you are mentioning and which is relevant.  

 However, we processed this implementation in the context of a POC and we are of course aware of the issue related to key generation. So much that, in a production setting, as discussed in the first round of revision, we would provide for the patients a proper generation key online interface doubled by a key registration with a material object, such as an USB cards to prevent from keys lost. This would enable us to benefit more broadly of the distributed nature of a “trustless” network. For sure, patients invention could still be possible but we believe that this would be a marginal phenomenon - besides, even in this case, since all the steps of consent would be timestamped on the blockchain, the entry barrier to fraud would be higher.

 In the far future we envision, we would think of a standardization of some key aspects of clinical trials methods whose critical steps would happen through blockchained transactions. For example, in the model of clinical trials platforms, such as www.clinicaltrials.gov, whose information is mandatory before the study starts, a good practice would be then for patients to register on such a platform, they would then have their own key identifiers.

**Proof of process**

*The authors should more clearly document the proof of process underlying their approach. The Chainscript examples (the code block and Figure 4–6) are a good start. However, Figure 4–6 are poor resolution and difficult to read. More discussion of the Chainscript format along with instructions to verify a process and timestamp are needed. For example, there is no example Chainscript JSON file provided with step-by-step instructions on how to verify or evaluate it. The Chainscript code block is littered with broken JSON syntax, so it's certainly an insufficient example. With more details, additional questions may arise. For example, is the 12-byte mapID (with a best-case attack complexity of 296) secure against preimage attacks? The manuscript doesn't appear to specify what hash algorithm is used.*

 - You can check any chainscript JSON file on the chainmap explorer : http://chainscript.io/#/chainmap-explorer.

 Now, let’s take as an example a sample Docchain Chain Map at http://chainscript.io/#/chainmap-explorer?application=docchain&mapId=59262a2eb446491fe9d48ecb

 If one click on any of the map, for example, the first one, titled “8671b7”, a JSON pops up, of which clock on the “JSON” tab, which will display the internal structure for that link. Going to the “evidence” or the “JSON” section, there is

   

   “state”: “COMPLETE”,

     “merkleRoot”: “21dd2f636a9d6b9cde1504e8fe413a65b1f2efc78d2bde1a09634cb55f0e2631”,

     “transactions”: {

       “bitcoin:testnet”: “b39d12a54226c976795dcc85c07bd0b02feffaf92a25f48cdb356fe3aa50fcfd”

     }

 Now if you look up the txId in the testnet blockexplorer, you will find the merkleroot in its OP_RETURN. For instance, you can try this

 https://www.blocktrail.com/tBTC/tx/b39d12a54226c976795dcc85c07bd0b02feffaf92a25f48cdb356fe3aa50fcfd

 - Beyond, you will find a detailed documentation of the step-by-step instructions you need in the ChainScript documentation. You’ll find an explanation of the overall logic of Chainscript and the Merkel tree data- structure on which it relies, a description of the JSON structure that holds the linked sets of blockchain transactions, and the source code enabling to verify the Merkel proof is valid and so that JSON structure is consistent. It has to be precised that the source code provided is adapted to a Node.js application.

 - The mapID is randomly generated, so there is no preimage attacks applicable to it. ehdiBesides, Chainscript is agnostic to the specific cryptographic scheme used to secure the process. E.g. if hashes of a certain data are stored inside the state, then the validation would equal checking hashes.

 Thus, for the verification of Chainmaps, it depends on the particular choice of data stored inside a segment, that is, the Chainscript does not enforce any specific format as the `link.state` is a freeform data. (As detailed here, a state is for example a concrete implementation of a notarizing process,  for example `uploading a file`, the state variable will store the uploaded name file, the user name or anything prescribed).

 - As for generating linkhash, it uses SHA256 of https://nodejs.org/api/crypto.html#crypto_class_hash. The process consists in converting the JSON into a string, which is done through a regular canonical-json module. This string is then hashed.

**Smart contracts**

*The manuscript is misleading regarding "smart contracts" and implies that the study proposes a smart contract enrollment protocol. Specifically:*

1. *Figure 1 includes a smart contract step in the workflow.*
2. *The abstract states "a blockchain core functionality, named Smart Contract, can help prevent clinical trial events not to happen in the right chronological order".*
3. *The introduction states "This makes it possible to build a Smart Contract that will be executed with the only condition that patients will only be included when the enrolment is complete".*

*As the authors admit in their response to my review of version 1, they "did not implement" anything related to smart contracts. Implementing a smart contract to manage enrollment is not trivial. Unless the authors actually implement such a contract, they should remove claims about its utility and applicability. Discussing smart contracts in the discussion would be justified, but it's misleading to suggest that the current proposal involves smart contracts.*

 We thank you for this remark, since smart contracts references appear to be far from clear. Indeed, the current state of the formulation can be misleading. The logic was to conclude the abstract section with drawing some perspectives, this is the reason for the sentence beginning with “In the future”.

 So we rewrote this part, suppressed the mention from the Introduction and we referred and detailed a bit more the discussion of this matter in the Discussion section.

**Overstated blockchain usage**

*In their response to my review of version 1, the authors clarify that the primary achievement of the study is to create a web-based consent workflow that makes it most natural and easy to perform the proper consent process. However, the study only minimally leverages the guarantees provided by cryptography and secure blockchains. For example, the study does not achieve trustless consent, whereby participant consent can be provided and verified in a decentralized manner without having to trust any other parties. However, in the abstract, the authors imply the trustless & decentralized aspects of Bitcoin apply to their consent process:*

**

*> This is a distributed technology that brings a built-in layer of transparency and traceability. Additionally, it removes the need for third parties, and gives participative control to the peer-to-peer users.*

 You’re absolutely right to affirm that we suppose indeed that there are stakeholders who have the root-permissions to create pairs keys are to be trusted. Indeed, the current process of clinical trials heavily relies on the existence of such stakeholders such as investigators, sponsors, regulatory agencies (FDA in the US, EMA in the EU, …) or IRBs. Depending on which stakeholders we trust, the generation of pair keys could be devoted to one of these stakeholders. For instance, if the objective is to insure mostly time stamping but investigators are trusted, the could generate the keys. If the system also wants to prevent forging consent by the investigator, then the keys could be generated by the sponsor or any other external party under the supervision of the sponsor, a regulatory agency or the IRB, and given to the patient with information documents. We thus believe our current implementation is directly usable for clinical trials as they are conducted today.

 Besides, we vigorously defend the point that patients should be more involved, and we envision that, in a near future, clinical trials will let a greater part to them, especially when it comes to deal with privacy concerns. Experiments, such as the “Compare” e-cohort we are currently leading in our department, enable precisely patients to be included in a fully online patient-centric study, so that it suits perfectly to host a blockchain layer as for the consent process. As detailed above, we believe that our implementation can be adapted to ensure users can generate their own sets of keys, then benefit from the distributed nature of the blockchain network, and by then getting closer to a trustless consent process.

 However, even we hope this kind of trials will become standards ones, this is not the case yet, so that our approach ensures a pragmatic usability of the current implementation of our POC by the stakeholders.

 Moreover, the idea of claiming what blockchain could bring, is also related to the deep outlook of numerous bias that entail research quality and clinical trials reproducibility. In fact many issues could benefit from the blockchain technology to be better controlled, as these are frequently related to a posteriori reconstruction or untraceable missing informations. Examples are the statistical analysis plan, the definition of outcomes, or inclusion and exclusion criteria, secondary effects traceability…

 From a “consent process“ point of view, we measure, even with the issue of this POC’s current stakeholder-sided authentication system, how much the incorruptible timestamping could drive benefits from the ground where current clinical trials are buit on : no traces other that handwritten for consents, no pairing between consents and protocol versionning... despite the issue related to key forgery, the process we are detailing prevails from a posteriori data manipulation, and this is not undoable because of the almost-inaccessible forgery of distributed ledgers. This participates of the transparency and traceability we mention.

 However, we understand that filling the gap from a POC to a production implementation requires a bit of work and so we downsized the claim of the article in order to take account of the issue you raised, that we of course identified and rejected to the implementation in a real setting. We warned the reader we did not yet implement a solution that fully exploit the distributed characteristics of a blockchain network.

*In reality, the only area where a blockchain was applied is for the Chainscript timestamping. I agree this timestamping is valuable for its ability to prevent retroactive consent forgery. However, it's insufficient to verify an actual participant's identify or consent. Foremost, the use of blockchain timestamping is not sufficient to justify the grandiose claims of blockchain relevance to clinical trial consent.*

**

*In other words, the proposed consent protocol would suffer little were all blockchains to immediately disappear. The blockchain is not essential to implement more automated, web-based, and reliable consent processes. Yet the study titles itself "blockchain protocols in clinical trials" and implies that blockchains are what allows "transparency and traceability of consent". The manuscript does not adequately differentiate between speculation and the actual ways in which the study leverages blockchain technology.*

**

*For me to consider approving this study, the authors would need to drastically reduce their claims regarding the benefits of blockchain usage for clinical trial consent applications. In addition, greater clarity and focus on the specifics of their proof of concept implementation would be necessary.*

 We purged the text from the claims that may be considered as  over-valued. For instance, we suppressed this sentence : “Additionally, it removes the need for third parties, and gives participative control to the peer-to-peer users’, “...a starting point to define a gold-standard of an open and secure informed consent collection process....”

 We enforced to distinguish more clearly prospective views from the current implementation. Especially, we rejected to the Discussion section evocation of future use of Smart Contract that we envisioned or the community-based aspects of blockchain technologies, since they are pertinent in a context where the authentication keys generation process happens on the patient side.

# Peer review: Referee response for version 1

**DOI:** 10.5256/f1000research.11349.r21311

**Published:** 2017-04-11

**Authors:** Daniel S. Himmelstein

**Affiliations:** 1 Systems Pharmacology and Translational Therapeutics, Perelman School of Medicine, University of Pennsylvania, Philadelphia, PA, USA

Benchoufi *et al.* propose and implement a method for notarizing participant consent for clinical trials using the Bitcoin blockchain. At a minimum, such an approach must accomplish two cryptographic objectives:

1. provide participants with a fraud-resistant method to irrefutably consent to the terms of a clinical trial.
2. provide clinical trial investigators with a method to retrospectively verify participant consent at a given point in time.

I agree with the authors that cryptographically notarizing consent would be a major advance. If possible, there would be strong incentives, both ethical and practical, for investigators to implement and regulatory agencies to mandate such an approach.

 By encoding a hash into the Bitcoin blockchain that derives from a "consent document", it is possible for investigators to timestamp a consent document and thereby retrospectively prove the existence of that consent document at a given point in time. However, I am not convinced that the study provides patients with a fraud-resistant, irrefutable method of consent. Specifically, I don't think the study provides a method for ensuring that a specific participant provided consent. In other words, can clinical trial investigators prove that a specific participant consented rather than just proving that some entity consented under the supposed digital identify corresponding to a specific participant?

 The study uses digital signatures to attest that a participant approved a specific consent form. The implementation relies on bitcoin private keys to provide digital signatures. The problem is that bitcoin addresses (which uniquely derive from a private key) are pseudonymous. For example, anyone with access to a consent form could create an unlimited number of bitcoin private keys and use these private keys to digitally sign the consent form. How does one prove that a bitcoin address solely belonged to a single participant?

 The generation of bitcoin private keys in this study occurs at https://git.io/vSPTE. I don't see anything in the code or manuscript that irrefutably links a participant to ownership of a given bitcoin address. Therefore, it appears to me that clinical trial investigators (or many other actors) could forge consent. In fact, the manuscript appears to concede this point with the statement:

 > Each of the to-be-enrolled users ***were assigned*** a private key in order to sign data and documents, and in practice this would be used to publish their signed consent.

 Linking digital identities to physical identities is difficult. However, this is a precondition to blockchain notarization of clinical trial consent. OpenPGP (the most established method for digital identity and signatures) relies on a web of trust model to link digital identities to physical identities. HTTPS relies on Certificate Authorities to link digital identities to organization identities.

 Since the manuscript does not sufficiently address how it links digital and physical identities, I dug a bit deeper into Stratumn, the underlying service provider. Stratumn is a French company, which aims to "secure processes between partners through blockchain technology." Stratumn focuses on "proof of process" using a JSON data structure called chainscript. I had difficulty uncovering the implementation details of Stratumn's proof of process, as much of the available online material focuses on the implications of the technology rather than the technology itself. The most helpful resource I found was the Epicenter Podcast #159 titled "Richard Caetano & Anuj Das Gupta: How Stratumn Secures Processes." This investigation did not answer how digital identities are linked to physical identities in Stratumn's services. The Stratumn proof-of-process white paper *does mention* identity verification under "Non-Repudiation of Source and Destination", stating:

 > Non-repudiation implies that the stakeholders of the information content of each and every step should not be able to deny their involvement with the steps representing their data through the digests. The tool we will use for this is Digital Signatures.

 > Both Alice and Bob need to be responsible to their respective steps in such a way they they can not repudiate their involvement if challenged. The record of their identities would be maintained by having the stakeholders digitally sign the digest of their move and then storing the signatures and public keys along with the digest in the step. The private keys will not be stored in the steps; each player hold hers separately and securely. Anyone who has access to the proof can use the public key verification to ascertain whether or not Alice or Bob can be held responsible for a step.

 > In this way we enable identity management and ownership in each and every step for the proof of a process to demonstrate the Who behind each and every step.

 Unfortunately, this description does not explain how digital identities are linked to physical identities. How does one know whether a digital signature is actually Alice or Bob's? Stratumn even provides a document detailing the clinical trial consent use case. However, this document does not provide an identity solution.

**Conclusion**

 I am marking my review as *Not Approved*. **If the authors can show that it is not trivial to forge a specific participant's consent, I would be happy to revisit my decision. However, absent a reliable method to link a digital identity to a participant's physical identity, there is little benefit to cryptographic notarization of clinical trial consent.** Such an approach is only as useful as its most vulnerable step. At a minimum, the authors need to identify the trusted parties related to participant identity.

**Minor points**

 The study could do a better job citing the relevant cryptographic literature. For example, the study cites neither the Bitcoin white paper nor the proof-of-process white paper. In addition, the study should consider referencing OriginStamp, OpenTimestamps, and Carlisle's 2014 blog post.

 Figures 2 & 3 are in French. I understand that it's important to show the consent form and interface as given to the participants. However, perhaps these should be supplements with English versions in the main text.

 The study states: "Smart contract: this is a contract that is algorithmically implemented and binds any change in the protocol to the patients’ consent seeking renewal." However, from my understanding the study does not propose any blockchain smart contracts. Instead, the Bitcoin blockchain is only used as a timestamping service.

**Positives**

 The study aims to replace trust with cryptography in medical research.

 The study makes its source code available under a permissive open source license on GitHub ( Zenodo archive).

 The study understands that directly writing every document hash to a secure & immutable blockchain will be cost prohibitive, and therefore it is necessary to "group transactions", i.e. write one transactions that attests to the existence of many chainscript hashes.

I have read this submission. I believe that I have an appropriate level of expertise to state that I do not consider it to be of an acceptable scientific standard, for reasons outlined above.

# Response

**Published:** 2017-04-19

**Authors:** mehdi benchoufi

**Affiliations:** Hotel-Dieu Hospital, Paris Descartes University, France

Dear reviewer, 

 Thank you for drawing our attention on the questions you raised. Our answer focuses mainly on the issue related to the binding between digital identities and physical entities. 

 We updated the revised version accordingly to all the questions you raised.  

**Target of the POC and digitale identities**

*POC as a response to FDA raised issues*

 Thank you for your constructive remarks. In fact, there are two main issues regarding the consent process. The first one is related to the quality of the process itself and the second one is related to the identity of the individual consenting. Monitoring of trials by the FDA has identified serious issues in about 10% of trials, major concerns being failure to obtain written informed consent, consent document not signed or not dated, missing pages in consent document provided to subjects,failure to re-consent when new information becomes available, use of expired, non-validated or unapproved forms (reference [3,4] in the manuscript and this link). In this POC study, we aimed at fighting these issues, where existing patients were included in a study in the presence of their physician or staff. In our implementation, we ensured that all the consent process was tracked in time all along the inclusion period, that the documents made available to the subjects were not corrupted, that they were in conformity with the current version of the protocol, and that (re-)consent was asked as many time as required. We however did not address the cases where consents were falsified by the investigating teams or where fake patients were invented for instance, which are more related to the second (identity) issue. This should be more explicit in the text, and has been emphasized in the revised manuscript.  

*Linking digital identities and physical entities*

 We then agree with the reviewer that the second issue is important in the context of a real clinical trial. This relates to the question of pairing digital identification with physical persons. In our implementation of the POC study, the link between a participant and his/her digital identity is done through his/her email address. A participant cannot simply generate any Bitcoin address and use it to sign, because the private key is created on the server by the agent (but not saved on the server), then is sent to the email address. So the only guarantee is that the participant had access to that email address. This is pretty light, but seemed to be satisfactory given the scope and focus of the POC. Moreover, in the setting of a real online consent process, there is no chance that a patient who would not effectively consent—for instance if there were some fraudulous operation registering him/her as a consenting subject—would actually participate to the study.

 However, in a production application we must implement a more secure mechanism, and we thank the reviewer again for outlining the possibilities. There are indeed several solution to secure the digital identity of participants.

 First, we can mention that KYC processes are now quite widespread and there are plenty of examples where digital identities are linked to physical entities or human beings in the context of sensitive data: in most western countries the fiscal administration allows online service for taxpayers to declare their income and proceed to related operations, banking services also provides their services online such as bank transfer, account consultation. All these examples have in common that users (taxpayers, bank clients) are given a physical material, often by sending a document by regular postal service, or by giving a first card or document by hand. These documents carry an identification number. This approach could be implemented in production.

 Second, even in regular off-line consent process, ensuring that the patient really signed the document is not without carrying problems. We have in mind that the patient must meet the medical doctor and receive by hand their consent document. So that, when they go back to the medical doctor with the signed document, there is no way to prove that it was signed by the patients himself or that the patient was not influenced at the time of signing the consent form.

 Regarding a possible implementation of our blockchained consent process in a real clinical trial, we can provide, at the moment the patient meets the doctor, authentication cards with an identification number and so strengthen the binding between the electronic signature id and the physical one. Let’s mention that some bitcoin companies also provide material keys, such as USB keys, that hold the cryptographic signature, which can be unlocked by an easy-to-remember code. This can be an excellent candidate to get stronger digital-physical binding. These issues are now covered in the revised discussion.

 Besides, we think that full online consent collection process raises more the issue to reach subjects that the one related to ensuring the targeted patient is really the one consenting.

*Patients invention*

 Going further, we can summarily refer to the extreme case of patients invention. Unfortunately, there is almost no way to prevent data invention. And there are some documented case in the medical literature, fortunately very rare, where clinical trial patients and all related clinical data were invented from end to end. Let’s notice here that, in the first implementation of our POC, we timestamped an extensive part of interactions between the subjects and the online email and web platform : time of the email sending, reception, time email was opened, the links being clicked on. We then validated the transactions into the blockchain, this way we could at least detect anomalies if such data appear grouped in time, so that it does not prevent from invention but higher the barrier to fraud.  

*Further technical improvements*

 On a more technical side, Stratumn’s technology, via proof-of-process, provide an implementation that would allow one to create an audit trail of the the different steps that happened until the participant provided enough evidence to convince the verifier of his/her identity, which is quite an upgrade to current KYC processes, because there would be a permanent record of what happened, and the process would impose rules that must be respected to the letter (by computer code) before moving to the next step. In short, this is not a complete out-of-the-box solution to linking digital identities, but Stratumn has the tools to build one. While implementing such a verification process with the technology is possible, this was beyond the scope of the POC.

**Smart Contracts**

 Indeed, we did not implement it in our setting, we meant to mention that Smart Contract can bind the modifications occurred in the protocol and some to be defined events on which parties can agree.  

 Besides, we did not detail or implement in the article all the advantages that can be derived from the algorithmic nature of the Smart Contracts: the way consent process is monitored, the way the related informations are shared between the stakeholders, the investigators and the IRB or building Smart contracts with the condition patients will only be included when the enrolment is complete or building one checking the whole consistency between the data and the blockchained proof of data, preventing from the whole hassle of gathering the documents and checking them by hand

**Figures**

 Indeed, some figures are in french and we complied to the F1000 policy that invited us to proceed so.

**Bibliography**

 We thank the reviewer for the precious remarks about bibliography and to have brought to the knowledge of the authors two of them, namely the one referred as `OriginStamp` and the Carlisle blogpost. We add and referenced them in the revised document.

 Best regards,

# Peer review: Referee response for version 1

**DOI:** 10.5256/f1000research.11349.r19560

**Published:** 2017-04-04

**Authors:** Mike Clarke

**Affiliations:** 1 Northern Ireland Methodology Hub, Centre for Public Health, Queen's University Belfast, Belfast, Ireland

This is an important article, worthy of publication in F1000Research. There are some places in the article where the writing could be tidied up (e.g. references 1 and 10 are the same) but my main comments relate to questions that prompted in my mind, which the authors might wish to address if they revise it:

 Proof of concept, blockchain and the study generally

1. Is the reported study a "proof of concept" for the use in a real trial, or simply a demonstration that blockchain can be used for a series of sequential "signings"? If the latter, had that not been shown previously?
2. Are there any plans to test this in a real trial, perhaps as a SWAT[1] and to include it in Trial Forge[2]?
3. How would this system be used if patients cannot get online personally?
4. How would the system cope if someone's email addresses changes? (I raise this because I am currently locked out of my Twitter account because the email I used to set it up is no longer active following my move away from that institution.)
5. Can you reflect more on the challenges of doing online trials?
6. Do you believe that this system will be applicable to all trials, a majority or a minority? You seem very enthusiastic about the use of this system and the article might benefit from the addition of more caution about its general applicability. For example, you write "we evoked a possible improvement in the enrolment rate, by empowering patients and granting them information and control over the enrolment phase." but say little of the possible negatives (such as concerns about security of the data (see below); fear or discomfort with technology; and whether empowerment might come more from the ability to talk to a human being about the trial and the consent process).
7. Who will ensure that blockchain is future proof? Might people need to print or export a copy of the electronic record for long-term storage?
8. Does blockchain allow for "workarounds" (e.g. to move to the next step without completing the previous one if for some reason this is necessary)?
9. What do you mean by "transparency" in relation to the new system? If a potential participant thinks this means that others can see that they gave their consent, might this discourage them from joining the study?
  Consent in general
10. How would this system cope with differences between the process for obtaining consent to take part in prospective research in different countries and cultures? For example, what if someone other than the patient might need to give consent?
11. Would patients be able to request that their ongoing consent is presumed without needing to be contacted again when there is a change in the trial? It might discourage patients from joining a trial if they are told that they will have to be contacted each time there is a change (especially if that change does not affect them personally).
12. What protocol changes should lead to new consent (e.g. should it only be those that directly affect the patient, or should it be those that might have influenced their decision to join?)
13. Would a patient need to be asked for their renewed consent if the change can no longer affect them? For example, if they have already completed treatment and are now on follow-up, do they need to be informed about changes in the evidence base about a side effect if they can no longer suffer that side effect?
14. Should patients be asked to consent again, or be asked if they want to withdraw? What assumption would be made if they do not reply?
15. Might it be worth discussing this new system in the context of other research into recruitment and retention (for example, as brought together in Cochrane Reviews [3,4,5,6]).
16. Is a lack of informed consent a source of bias (or might it be closer to the "truth" if patients don't realise that they are being studied) or bad ethical practice?
17. Might it be worth discussing the double standards of needing written consent for someone to receive a treatment in a trial but not needing it if they are given the treatment as part of "routine practice"?
18. How important is "written consent"? Is this unfair on difficult to reach populations who struggle to read or write?
  Electronic consent
19. How would you ensure that the appropriate person "signed" the consent form if you do not see them do so? Is it easier to submit someone's electronic key, than to forge their signature?
  Security
20. Might patients' concerns over the security of their data and the importance of confidentiality make them cautious about joining a trial if they had to use this system? How worried might they be because of news stories about data from banks and other supposedly secure systems being hacked and leaked?
21. Might it be worth writing something about how patients may think that paper consent forms locked in a filing cabinet are more difficult to access and make available to everyone online, than documents that are already available to the research team from anywhere on the internet.
  Language
22. The words "subject" and "participant" are used to refer to people who take part in trials. I prefer to avoid "subject".

I have read this submission. I believe that I have an appropriate level of expertise to confirm that it is of an acceptable scientific standard.

## References

[1] ClarkeMSavageGMaguireLMcAneneyH: The SWAT (study within a trial) programme; embedding trials to improve the methodological design and conduct of future research. *Trials*.2015;16(S2) : 10.1186/1745-6215-16-S2-P209 10.1186/1745-6215-16-S2-P209

[2] TreweekSAltmanDBowerPCampbellMChalmersICottonSCraigPCrosbyDDavidsonPDevaneDDuleyLDunnJElbourneDFarrellBGambleCGilliesKHoodKLangTLittlefordRLoudonKMcDonaldAMcPhersonGNelsonANorrieJRamsayCSandercockPShanahanDSummerskillWSydesMWilliamsonPClarkeM: Making randomised trials more efficient: report of the first meeting to discuss the Trial Forge platform. *Trials*.2015;16(1) : 10.1186/s13063-015-0776-0 10.1186/s13063-015-0776-0 PMC447533426044814

[3] TreweekSLockhartPPitkethlyMCookJAKjeldstrømMJohansenMTaskilaTKSullivanFMWilsonSJacksonCJonesRMitchellED: Methods to improve recruitment to randomised controlled trials: Cochrane systematic review and meta-analysis. *BMJ Open*.2013;3(2) : 10.1136/bmjopen-2012-002360 10.1136/bmjopen-2012-002360 23396504PMC3586125

[4] BruetonVCTierneyJStenningSHardingSMeredithSNazarethIRaitG: Strategies to improve retention in randomised trials. *Cochrane Database Syst Rev*.2013; 10.1002/14651858.MR000032.pub2 MR000032 10.1002/14651858.MR000032.pub2 24297482PMC4470347

[5] SynnotARyanRPrictorMFetherstonhaughDParkerB: Audio-visual presentation of information for informed consent for participation in clinical trials. *Cochrane Database Syst Rev*.2014; 10.1002/14651858.CD003717.pub3 CD003717 10.1002/14651858.CD003717.pub3 24809816PMC6599866

[6] GilliesKCottonSCBrehautJCPolitiMCSkeaZ: Decision aids for people considering taking part in clinical trials. *Cochrane Database Syst Rev*.2015; 10.1002/14651858.CD009736.pub2 CD009736 10.1002/14651858.CD009736.pub2 26613337PMC8725643

# Response

**Published:** 2017-04-19

**Authors:** mehdi benchoufi

**Affiliations:** Hotel-Dieu Hospital, Paris Descartes University, France

**Proof of concept, blockchain and the study generally**

 Dear reviewer, 

 Here are some responses to the questions that were raised. Besides, thank you for your remark related to the bibliography and the reference duplicate. 

 1. *Is the reported study a "proof of concept" for the use in a real trial, or simply a demonstration that blockchain can be used for a series of sequential "signings"? If the latter, had that not been shown previously?*

 In the idea of establishing all the consent process in real conditions, we claim it is a proof of concept. We developed a complete and realistic set of interactions between fake patients and stakeholders, and we paired consent status and protocol revision through blockchain held by a single master document accounting for the whole process.

 2. *Are there any plans to test this in a real trial, perhaps as a SWAT[1] and to include it in Trial Forge[2]?*

 For the moment, we have no specific plan to implement in a real trial but our POC is precisely a preparation to go further to a real setting.

 We have no expertise in SWAT or in using Trail Forge platform, but it should comply with no difficulty with our implementation.

 3. *How would this system be used if patients cannot get online personally?*

 This is more a problem related to online consent than a blockchain related question. In situations where patients cannot get online personally, then the medical doctor should provide the consent form to the subjects by hand. Besides, let's indicate that in most countries, the written consent is legally mandatory.

 There are other situations where the online access is not possible, related for example to disabilities, then either the legal representative should be given access to the online consent form, or written consent should be sought.

 4. *How would the system cope if someone's email addresses changes? (I raise this because I am currently locked out of my Twitter account because the email I used to set it up is no longer active following my move away from that institution.)*

 In a real clinical trial, we would set multiple ways to reach a patient : digitally, phone number, postal mail. For sure, email is not sufficient, and we would use a stronger identifying system than email, i.e. material objects storing cryptographic keys, such as a USB key. We could also build a Smart Contract triggered by a destination email error callback. Then, when this condition is met, Smart Contract would cause the other reaching methods to be proceeded. Since Smart Contract are pieces of code, this automatized processed can be customized at will.

 However, although this is not the situation you are pointing to, this procedure may find a limit: there is no way to distinguish between the situation where an email has been sent without response, and the one where the email is still maintained by the institution with no access anymore for the previous account holder. But, we think this very case looks quite exceptional.

 5. *Can you reflect more on the challenges of doing online trials?*

 There are some challenges regarding online trials:

 - people that are not in a condition to access an online platform: severe conditions, lack of consciousness, learning disability, no access to internet or people not friendly to online tools

 - the current state of technologies do not allow interventional trials. However, the explosion of IoT, miniaturization could lead to imagine some specific interventional trials to be conducted online. In this respect, we mention the existence of blockchain systems specifically dedicated to connected objects.

 - ensuring that the person consenting is effectively the one pretending to be: this should lead to use a strong identifier, and at minimum use KYC process (Know Your Customer) implemented to bind digital identities to physical entities in the case of sensitive data. Fiscal administration, Banks makes use of that.

 It is worth noting that from a blockchain point of view, the email is by no mean a method to identify. In our settings, we generate identifiers for each patient which consists in complex cryptographic strings. It would be possible in addition of these informations stored in the local machine of the subject, to duplicate this information on a USB key or identifier cards on the model of KYC procedure. Let's state that there are some companies providing material supports to keep the blockchain id's.

 6. *Do you believe that this system will be applicable to all trials, a majority or a minority? You seem very enthusiastic about the use of this system and the article might benefit from the addition of more caution about its general applicability. For example, you write "we evoked a possible improvement in the enrolment rate, by empowering patients and granting them information and control over the enrolment phase." but say little of the possible negatives (such as concerns about security of the data (see below); fear or discomfort with technology; and whether empowerment might come more from the ability to talk to a human being about the trial and the consent process).*

 Indeed, blockchain is not without carrying some issues. Our point was to put in a perspective a trend. Every new technological gap raises some fears, as the internet at its very beginning, but the overall trend is that usages have imposed. It is worth noting that users shape also technologies and their fear pushes the improvement of technologies conducting these to take more account of their needs and apprehensions.

 Besides, our idea is not to rely on a technology by itself but to complete the blockchain network ability to ensure data consistency by a strong human support at any step of the clinical trial, first and foremost the consent process.

 Besides, from the data security point of view, we positively think that it is much better ensured by blockchain-like technologies than the one currently used : first, because of the strong crypto-oriented transaction validation, second the distributed nature of the network prevent from the "single point of failure" problem related to centralized data collection. By the way, we can take the Bitcoin network as an example, which carries sensitive money data and which is proving to be resistant for almost ten years.

 7. *Who will ensure that blockchain is future proof? Might people need to print or export a copy of the electronic record for long-term storage?*

 Blockchain consist of a network of peers, anybody involved in the network storing in his computer the archive of all the history of transactions, i.e. the public ledger. So that, even if the network had to fail for one or other reason, any single node can restore the last state of the network. Moreover, if necessary, depending on the design of the blockchain implementation, we might enable the only stakeholders or if we want any participant, to export and print the copy of electronic transactions.

 We may have in mind that data stored are "proof of data" that can be checked to match the true data on any dedicated public website.    

 8. *Does blockchain allow for "workarounds" (e.g. to move to the next step without completing the previous one if for some reason this is necessary)?*

 Yes, one core functionality of blockchain technologies, is Smart Contract. They allow to write algorithmically any set of conditions that modules the execution of some instructions.

 But, we stress the fact the system can be "fault-tolerant" but there are no "roll-back" possibility. So, suppose that someone stores some informations but has mistaken, then he'll be able to add the new corrected information (that's what we can call a "fork"), but the first errored information is still accessible in the blockchain.

 9. *What do you mean by "transparency" in relation to the new system? If a potential participant thinks this means that others can see that they gave their consent, might this discourage them from joining the study?*

 We mean by transparency that the rules are clear for anybody taking part in the process. This is the very role of the Smart Contract we already mentioned.

 Besides, the perimeter of the people sharing the data can be controlled : we can decide that data can be shared by the only stakeholders, or decide that participants will be able to share access to their data with persons of trust. All this fine-grained control can be powered on the top of blockchain.

**Consent in general**

 10. *How would this system cope with differences between the process for obtaining consent to take part in prospective research in different countries and cultures? For example, what if someone other than the patient might need to give consent?*

 Differences between cultures, countries need to be tackled one by one. So that we need true implementation of clinical trials to face with those kinds of issues. I don’t think there is a one general answer.

 Online tools can help orienting the participant regarding to their specific situation. Moreover, we believe chat support should be provided in order to take account of specific situations  

 11. *Would patients be able to request that their ongoing consent is presumed without needing to be contacted again when there is a change in the trial? It might discourage patients from joining a trial if they are told that they will have to be contacted each time there is a change (especially if that change does not affect them personally).*

 Indeed, there is no need to overwhelm patients with a flood of informations. However, from a general point of view, patients association often complain about lack of informations regarding the clinical trial progress, so that some calibrated informations can be delivered conveniently to the patients.  

 Moreover, in case of a major change in the protocol, they should be specifically targeted to get an email asking to consent again.  

 12. *What protocol changes should lead to new consent (e.g. should it only be those that directly affect the patient, or should it be those that might have influenced their decision to join?)*

 There is heavy litterature about this. We refer it in the article: [10,11,12] in our bibliograhy  and we mentionned these links detailing the protocol changes that should lead to renew the consent: http://www.irb.pitt.edu/sites/default/files/reconsent guidance.pdf; http://www.mayo.edu/research/documents/29-re-consent-or-notification-of-significant-new-findingspdf/doc-10027714; http://www.yale.edu/hrpp/policies/documents/Reconsentingguidance.pdf)

 13. *Would a patient need to be asked for their renewed consent if the change can no longer affect them? For example, if they have already completed treatment and are now on follow-up, do they need to be informed about changes in the evidence base about a side effect if they can no longer suffer that side effect?*

 Yes, these kind of new informations should be given to patients and require to ask for a renewed consent again, even if they are not supposed to suffer this side effect thereafter.   

 14. *Should patients be asked to consent again, or be asked if they want to withdraw? What assumption would be made if they do not reply?*

 If patients want to withdraw, they should not be asked again.

 When patients do not reply, and after ensuring by other means (emails, mail, phone call if available) that they do not provide an answer, then they should be considered has consenting because they already gave their consent.

 Besides, this kind of situation can be advantageously scheduled in a Blockchain Smart Contract, that can be adapted to local legal contexts.

 15. *Might it be worth discussing this new system in the context of other research into recruitment and retention (for example, as brought together in Cochrane Reviews [3,4,5,6]).*

 Indeed, we might implement some Smart Contracts, checking the recruitment and retention process. So Cochrane reviewers could have an insight about whether this process was done conformally to standard procedures. However, we notice that the code of the Smart Contract should also be reviewed, so that an experienced developer should be required.

 16. *Is a lack of informed consent a source of bias (or might it be closer to the "truth" if patients don't realise that they are being studied) or bad ethical practice?*

 Lack of informed consent is for sure a bad ethical practice, in a strict contradiction to Helsinki declaration, Nuremberg declaration and good clinical practices.

 Regarding the matter of generalisability bias, the latter is more related to the setting of inclusive criteriae than related to consent.

 17. *Might it be worth discussing the double standards of needing written consent for someone to receive a treatment in a trial but not needing it if they are given the treatment as part of "routine practice"?*

 In any case, the participant to a clinical trial must to sign the consent form, even he is already taking the medication for which the consent form is seeking his/her consent. At this occasion, the patients may be informed of actual informations related to the treatment, for example new side effects of a drug.

 18. *How important is "written consent"? Is this unfair on difficult to reach populations who struggle to read or write?*

 The written status of the consent by opposition of the electronic has not by itself more credit. However, depending on the local legal context, “written consent” is mandatory.

 Besides, collecting consent of people with reading, writing or learning disabilities needs care. The person collecting the consent must assess the ability of the person to understand correctly the informations and to make a decision. The information must be given orally and to the legal representative if any. Some ethics committees allow the mediation of families or other supports at this stage.

**Electronic consent**

 19. *How would you ensure that the appropriate person "signed" the consent form if you do not see them do so? Is it easier to submit someone's electronic key, than to forge their signature?*

 In this POC, we generated cryptographic keys for each patient. So, in this design indeed, we can't ensure that the person consenting is the person he or she pretends to be. This can be improved in different manner.

 At least, using KYC standard procedures, i.e. doubling the electronic identification by one related to a physical object holding some number code. One step further would be to provide patients with an objects storing USB key storing the cryptographic signature and unlocked by a easy-to-remember id.

**Security**

 20. *Might patients' concerns over the security of their data and the importance of confidentiality make them cautious about joining a trial if they had to use this system? How worried might they be because of news stories about data from banks and other supposedly secure systems being hacked and leaked?*

 We refer to the question 6 on the notion of “single point of failure”, which answers at some level the raised issue.

 In any case, patients are very sensitive to the security and the privacy of their data and this system addresses precisely this issue. Indeed, the decentralized structure of the blockchain, the involvement of anyone as a peer on the network, the ability to finegrain the data sharing perimeter, allows more control of the patients over the data workflow.

 However, we are conscious that this system is very new and needs some pedagogical support. Anyway, Bitcoin is now a widespread, trusted electronic currency, available on payment platform of a wide range of websites such as Amazon, Apple’s App Store. An implementation of such processes in a real clinical trial should be the occasion to add different media support : documents, videos in order to inform patient of the benefit of using such technologies .

 For the second part of the question, see the response to question 6. of the present reviewing question list.     

 21. *Might it be worth writing something about how patients may think that paper consent forms locked in a filing cabinet are more difficult to access and make available to everyone online, than documents that are already available to the research team from anywhere on the internet.*

 Absolutely it might be very interesting.

**Language**

 22. The words "subject" and "participant" are used to refer to people who take part in trials. I prefer to avoid "subject".

 We substituted the usage of "subjects" by "participants" in the revised document.

 Best regards,
